# Pitfalls in Fractal Time Series Analysis: fMRI BOLD as an Exemplary Case

**DOI:** 10.3389/fphys.2012.00417

**Published:** 2012-11-15

**Authors:** Andras Eke, Peter Herman, Basavaraju G. Sanganahalli, Fahmeed Hyder, Peter Mukli, Zoltan Nagy

**Affiliations:** ^1^Institute of Human Physiology and Clinical Experimental Research, Semmelweis UniversityBudapest, Hungary; ^2^Diagnostic Radiology, Yale UniversityNew Haven, CT, USA; ^3^Biomedical Engineering, Yale UniversityNew Haven, CT, USA

**Keywords:** fractals, monofractals, multifractals, time series analysis, numerical testing, fMRI BOLD, brain

## Abstract

This article will be positioned on our previous work demonstrating the importance of adhering to a carefully selected set of criteria when choosing the suitable method from those available ensuring its adequate performance when applied to real temporal signals, such as fMRI BOLD, to evaluate one important facet of their behavior, fractality. Earlier, we have reviewed on a range of monofractal tools and evaluated their performance. Given the advance in the fractal field, in this article we will discuss the most widely used implementations of multifractal analyses, too. Our recommended flowchart for the fractal characterization of spontaneous, low frequency fluctuations in fMRI BOLD will be used as the framework for this article to make certain that it will provide a hands-on experience for the reader in handling the perplexed issues of fractal analysis. The reason why this particular signal modality and its fractal analysis has been chosen was due to its high impact on today’s neuroscience given it had powerfully emerged as a new way of interpreting the complex functioning of the brain (see “intrinsic activity”). The reader will first be presented with the basic concepts of mono and multifractal time series analyses, followed by some of the most relevant implementations, characterization by numerical approaches. The notion of the dichotomy of fractional Gaussian noise and fractional Brownian motion signal classes and their impact on fractal time series analyses will be thoroughly discussed as the central theme of our application strategy. Sources of pitfalls and way how to avoid them will be identified followed by a demonstration on fractal studies of fMRI BOLD taken from the literature and that of our own in an attempt to consolidate the best practice in fractal analysis of empirical fMRI BOLD signals mapped throughout the brain as an exemplary case of potentially wide interest.

## Introduction

Fractality (Mandelbrot, 1967, 1980, 1985; Bassingthwaighte et al., 1994; Gouyet, 1996; Eke et al., 2002), – in addition to deterministic chaos, modularity, self-organized criticality, “small word” network-connectivity – by now has established itself as one of the fundaments of complexity science (Phelan, 2001) impacting many areas including the analysis of brain imaging data such as fMRI BOLD (Zarahn et al., 1997; Thurner et al., 2003; Maxim et al., 2005; Raichle and Mintun, 2006; Fox et al., 2007; Razavi et al., 2008; Wink et al., 2008; Bullmore et al., 2009; Herman et al., 2009, 2011; Ciuciu et al., 2012).

The interest in fractal analysis accelerated the development of the new paradigm beyond a rate when the new – essentially mathematical or physical (i.e., statistical mechanics) – knowledge could be consolidated, their tools thoroughly evaluated and tested before being put to wide-spread use in various fields of science; typically beyond the frontiers of mathematics. The lack of an in-depth understanding of the implications of the methods when applied to empirical data, often generated conflicting results, but also prompted efforts at making up for this deficiency. Early, with the migration of the fractal concept from mathematics to various fields of science like physiology, the groups of Bassingthwaighte (Bassingthwaighte, 1988; Bassingthwaighte et al., 1994) and Eke et al. (1997) realized the need to adopt a systematic approach in developing needed analytical and testing frameworks to characterize and evaluate various monofractal time series methods (Bassingthwaighte and Raymond, 1994, 1995; Caccia et al., 1997; Eke et al., 2000, 2002). Eke and coworkers demonstrated that conscious and precise monofractal time series analysis could only be done when one has an *a priori* concept of the nature of the observed signals. They introduced the dichotomous fractional Gaussian noise (fGn)/fractional Brownian motion (fBm) model of Mandelbrot and Ness (1968) as the basis of monofractal time series analysis (Eke et al., 2000, 2002) and offered a strategy for choosing tools according to a proven selection criteria (Eke et al., 2000). Given the continuing advance in the fractal field and in sync with the increasing awareness to avoid potential pitfalls and misinterpretation of results in various forms of fractal analyses (Delignieres et al., 2005; Gao et al., 2007; Delignieres and Torre, 2009; Marmelat and Delignieres, 2011; Ciuciu et al., 2012), in this article we apply our evaluation strategy to multifractal tools, and characterize their most widely used implementations. Our motivation in doing so stems from the potentials of fMRI BOLD multifractal analysis in revealing the physiological underpinnings of activation-related change in scaling properties in the brain (Shimizu et al., 2004).

fMRI BOLD (Ogawa et al., 1990, 1993b; Kwong et al., 1992; Bandettini, 1993) has been selected as an exemplary empirical signal in our demonstrations, because its impact on contemporary neuroscience (Fox and Raichle, 2007). The human brain represents the most complex form of the matter (Cramer, 1993) whose inner workings can only be revealed if signals reflecting on neuronal activities are recorded at high spatio-temporal resolution. One of the most powerful methods, which can record spatially registered temporal signals from the brain, is magnetic resonance imaging (MRI; Lauterbur, 1973). The MRI scanner can non-invasively record a paramagnetic signal (referred to as blood oxygen level dependent, BOLD; Ogawa et al., 1990, 1993a) that can be interpreted as the signature of the functioning brain via its metabolic activity continuously modulating the blood content, blood flow, and oxygen level of the blood within the scanned tissue elements (voxels). Recently, a rapidly increasing volume of experimental data has demonstrated that BOLD is a complex signal, whose fractality – if properly evaluated – can reveal fundamental properties of the brain among them the so called “intrinsic or default mode” of operation that appears complementing the stimulus-response paradigm in the understanding the brain in a powerful way (Raichle et al., 2001). We hope, our paper could contribute to this major effort from the angle of consolidating some relevant issues concerning fractal analysis of fMRI BOLD.

## Concept of Fractal Time Series Analyses

### Monofractals

All fractals are self-similar structures (mathematical fractals in an exact, natural fractals in a statistical sense), with their fractal dimension falling between the Euclidian and topologic dimensions (Mandelbrot, 1983; Eke et al., 2002). When self-similarity is anisotropic, the structure is referred to as self-affine; a feature, which applies to fractal time series (Mandelbrot, 1985; Barabási and Vicsek, 1991; Eke et al., 2002), too. Statistical fractals cannot be described comprehensively by descriptive statistical measures, as mean and variance, because these do depend on the scale of observation in a power law fashion:

(1) $$\frac{\mu_{2}}{\mu_{1}}={\left(\frac{s_{2}}{s_{1}}\right)}^{\varepsilon },$$

where μ_1_, μ_2_ are descriptive statistical measures, and *s*_1_, *s*_2_ are scales within the scaling range where self-affinity is present, and ε is the power law scaling exponent. From this definition a universal scale-free measure of fractals can be derived:

(2) $$D=-\underset{s\to 0}{\lim}\left(\inf\frac{\log\left(N\left(s\right)\right)}{\log\left(s\right)}\right).$$

*D* is called capacity dimension (Barnsley, 1988; Liebovitch and Tóth, 1989; Bassingthwaighte et al., 1994), which is related but not identical to the Hausdorff dimension (Hausdorff, 1918; Mandelbrot, 1967), *s* is scale and *N*(*s*) is the minimum number of circles with size *s* needed to cover the fractal object to quantify its capacity on the embedding dimensional space (it corresponds to μ in Eq. 1). For fractal time series, the power law scaling exponent ε is typically calculated in the time domain as the Hurst exponent (*H*), or in the frequency domain as the spectral index (β). *H* and *D* relate (Bassingthwaighte et al., 1994) as:

(3) H=2-D.

Further, β can also be obtained from *H* as (*H* − 1)/2 for fGn and (*H* + 1)/2 for fBm processes (Eke et al., 2000).

### Multifractals

While *D* does not vary along a monofractal time series, it is heterogeneously distributed along the length of a multifractal signal.

This phenomenon gave rise to the term “singular behavior,” as self-affinity can be expressed by differing power law scaling along a multifractal time series, *X*_i_ as:

(4) $$X_{i+\Delta i}-X_{i}\propto {\left|\Delta i\right|}^{h\left(i\right)}\text{,}$$

where *h* is the Hölder exponent defining the degree of singularity at time point, *i*. Calculating the fractal dimension for each subsets of *X_i_* of the same *h*, one obtains the singularity spectrum, *D*(*h*) (Mandelbrot spectrum), which describes the distribution of singularities (Frisch and Parisi, 1985; Falconer, 1990; Turiel et al., 2006).

(5) $$D\left(h\right)=\frac{\log\left(\rho \left(h\right)∕\rho \left(h_{\max}\right)\right)}{\log s_{\min}},$$

where *h*_max_ is the Hölder exponent corresponding to maximal fractal dimension, *s*_min_ is the finest scale corresponding to Hölder trajectory, and ρ(*h*) is the distribution of singularities.

The singular behavior of a multifractal is a local property. Separation of the singularities can be difficult, given the finite sampling frequency of the signal of interest (Mallat, 1999). Thus, in contrast with monofractality, a direct evaluation of multifractality is a demanding task in terms of the amount of data and the computational efforts needed, which can still not guarantee precise results under all circumstance.

**Figure 1:**
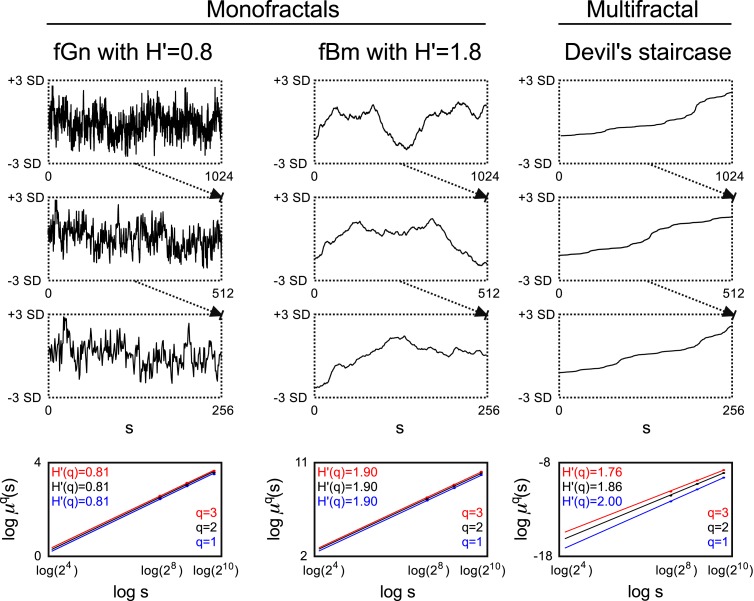
**Monofractal and multifractal temporal scaling**. Three kinds of fractals are shown to demonstrate scale-free property of these structures: a stationary monofractal (fractional Gaussian noise), a non-stationary monofractal (fractional Brownian motion), and a multifractal (Devil’s staircase with weight factors *p*_1_ = *p*_3_ = 0.2, *p*_2_ = 0.6). Every fractal is self-similar: fGn and fBm in a statistical sense (as in empirical structures and processes where fractality is manifested in equal distributions, only) and Devil’s staircase in an exact manner (as self-similar structuring in mathematical, i.e., ideal fractals is exact). For fractals, descriptive statistical measures [for example mean, variance, fluctuation (*F_q_*) etc.] depend on the corresponding scale in a power law fashion. Thus as a scale-free descriptor, the extended Hurst exponent (*H*′) is calculated as a slope of regression line between the logarithms of the scale (*s*) and *F_q_* (For an explanation of *H*′, see main text). The obtained slopes for different magnifications of the time series [here with the order of *q* = (1, 2, 3), which is the order of moment of the used measure] are the same for monofractals and different for multifractals, demonstrating that power law scaling behavior is a global property of monofractals, while it is a local property of multifractals. Accordingly, note that slopes in the bottom left and middle panel are the same, while in the right panel they indeed differ. For further details, see main text.

With the aid of different moments of appropriate measure, μ, a set of equations can be established to obtain the singularity spectrum, which is a common framework exploited by multifractal analysis methods referred to as *multifractal formalism* (Frisch and Parisi, 1985; Mandelbrot, 1986; Barabási and Vicsek, 1991; Muzy et al., 1993). Using a set of different moment orders, one can determine the scaling behavior of μ*^q^*, yielding the generalized Hurst exponent, *H*(*q*) (Barunik and Kristoufek, 2010; See Figure 1):

(6) $$\left\langle \mu^{q}\left(s\right)\right\rangle \propto {s^{q\cdot }}^{H\left(q\right)}.$$

On the right side of Eq. 4 Δ*i* corresponds to scale, *s*, on the right side of Eq. 6. Using the partition function – introduced in context of Wavelet Transform Modulus Maxima (WTMM) method – singularities are analyzed globally for estimating the (multi)scaling exponent (Mallat, 1999):

$$\begin{array}{llll} & Z\left(s,q\right)=\overset{N\left(s\right)}{\underset{k=1}{\sum }}\mu_{i}^{q}\left(s\right) & & \text{(7)}\\ & \tau \left(q\right)=\underset{s\to 0}{\lim}\;\inf\frac{\log Z\left(s,q\right)}{\log s}, & & \text{(8)} \end{array}$$

where τ(*q*) can be also expressed from *H*(*q*) (Kantelhardt et al., 2002) as:

(9) $$\tau \left(q\right)=q\cdot H\left(q\right)-D_{\text{T}},$$

where *D*_T_ is the topological dimension, which equals 1 for time series.

The generalized fractal dimension can also describe the scale-free features of a multifractal time series:

(10) $$D\left(q\right)=\frac{\tau \left(q\right)}{q-1}=\frac{q\cdot h\left(q\right)-1}{q-1}.$$

**Figure 2:**
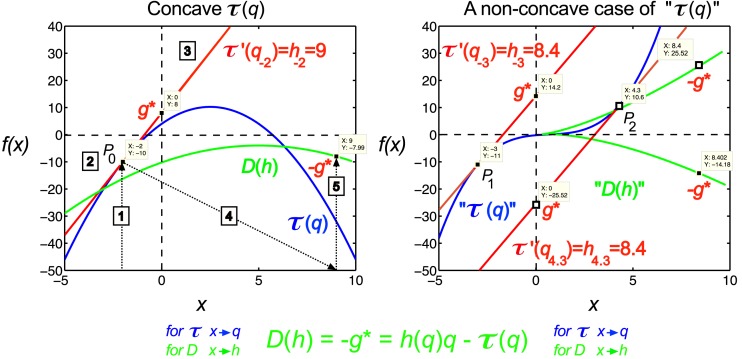
**Legendre transform**. It is known that singularity spectrum, *D*(*h*), has a concave shape, and provided that τ(*q*) is also a concave function, they can be explicitly transformed into each other via the Legendre transform (Bacry et al., 1993). Legendre transform takes a function, in our case τ(*q*) and produces a function of a different variable, *D*(*h*). The Legendre transform is its own inverse and uses minimization as the basis of the transformation process according to Eq. 12. If minimization cannot be achieved, the transformation would fail. On the left a real (concave), on the right a non-concave case for τ(*q*) is shown. A simple concave function, *f*(*x*) = −*x*^2^ + 5*x* + 4 (shown in blue) is used for modeling τ(*q*). If *f*(*x*) is differentiable, hence a tangent line (shown in red) can be taken at point of *P*_0_ (*q*_0,_ τ_0_) with a slope τ′(*q*), then g*(*q*_0_) is the *y*-intercept, (0, g*), and −g* is the value of the Legendre transform (See Eq. 11). Maximization at (*q*_0,_ τ_0_) is valid since for any other point on the blue curve, a line drawn through that point with the same slope as the red line will yield a τ_0_-intercept below the point (0, g*), showing that g* is indeed obtained as a boundary value (maximum), thus the transformation for *D*(*h*) would also yield a single boundary value (minimum) on the green curve as *D*(*h*) = −g* = τ′(*q*)*q*−τ(*q*). Steps of the transformation process are shown (1) select *q*, (2) read τ(*q*), (3) take a tangent line at (*q*, τ) and determine its slope, *h* = τ′(*q*), (4) select *h*, (5) determine *D*(*h*) using the above equation; repeat for the set. On the right side, a non-concave function is shown (blue) for demonstrating a case, when due to the non-concave shape of τ(*q*) the shape of the transformed function, *D*(*h*), does not yield a realistic singularity spectrum given that in this case the transform by failing on minimization is poorly behaved yielding ambiguous values.

The singularity spectrum, *D*(*h*), can be derived from τ(*q*) with Legendre transform (Figure 2), via taking

(11) $$h=\tau^{\prime }\left(q\right),$$

the slope of the tangent line taken at *q* for τ(*q*), and yielding

(12) $$D\left(h\right)=\underset{q}{\inf}\left(qh-\tau \left(q\right)\right),$$

that when evaluated gives the negative of the intercept at *q* = 0 for the tangent line (See Figure 2).

**Figure 3:**
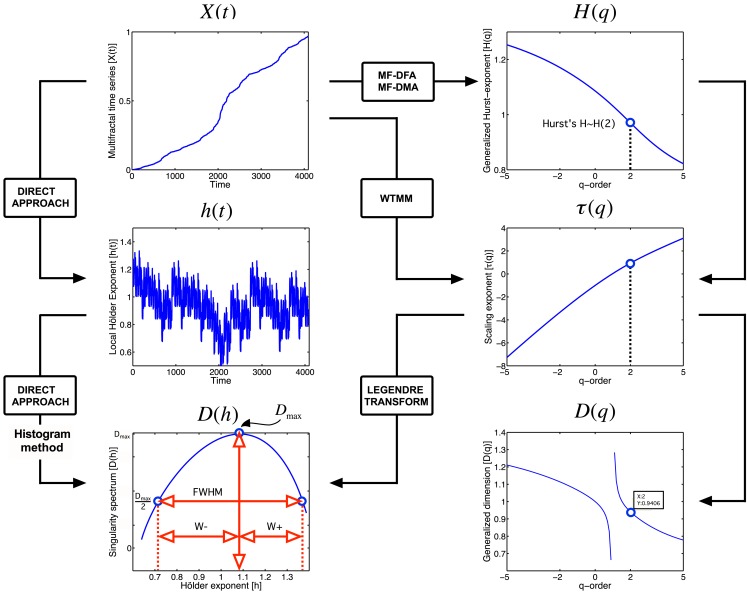
**Approaches to multifractal analyses**. Direct approach of multifractal analysis means exploiting the local power law scaling behavior to obtain local Hölder exponents (Eq. 4), from which the Mandelbrot spectrum is calculated with histogram method (Falconer, 1990; Eq. 5). Indirect approaches shown here (MF-DFA, multifractal detrended fluctuation analysis; MF-DMA, multifractal detrended moving average; WTMM, Wavelet Transform Modulus Maxima) estimates the scaling exponent, τ as a function of *q*. It is worth to note, that this is carried out differently for MF-DFA, MF-DMA (Eq. 9), and for WTMM (Eq. 8). From τ(*q*), the Mandelbrot spectrum can be obtained with the application of the Legendre transform, while its relation to generalized fractal dimension *D*(*q*) is given by Eq. 10. Singularity spectrum, *D*(*h*), is an important endpoint of the analysis. The spectrum is concave and has a nearly parabolic shape with a maximum identified by the capacity dimension at *q* = 0 (Mallat, 1999; Shimizu et al., 2004; Ihlen, 2012). Please note that some of its measures (*FWHM*, *D*_max_, *W* + , *W*−) can be used to calculate meaningful combined parameters (such as *P*c, and *W* in Eqs 13 and 14, respectively) with potential in correlating with key features of fMRI BOLD time series.

Natural signals have a singularity spectrum over a bounded set of Hölder exponents, whose width is defined by [*h*_−∞_, *h*_+∞_] (Figure 3).

A combination parameter, *P*_c_, can be calculated (definitions on Figure 3) to facilitate the separation of time series characteristics (Shimizu et al., 2004), which can aid the exploration of the physiological underpinnings, too.

(13) $$P_{c}=\frac{h_{\max}}{D_{\max}}\cdot FWHM.$$

A similar parameter is *W* (Wink et al., 2008) calculated as

(14) $$W=\frac{W+}{W-}.$$

## Implementation of Fractal Time Series Analyses

Implementation of concepts in reliable algorithms is a critical task, as stationary and non-stationary signals require different methods when analyzed for their fractality. For a stationary signal the probability distribution of signal segments is independent of the (temporal) position of the segment and segment length, which translates into constant descriptive statistical measures such as mean, variance, correlation structure etc. over time (Eke et al., 2000, 2002).

Accordingly, signals can be seen as realizations of one of two temporal processes: fBm, and fGn (Eke et al., 2000). The fBm signal is non-stationary with stationary increments. An fBm signal, *X_i_*, is self-similar in that its sampled segment *X_i,n_* of length *n* is equal in distribution with a longer segment *X_i,sn_* of length *sn* when the latter is rescaled (multiplied) by *s^-H^*. This means that every statistical measure, *m_n_*, of an fBm time series of length *n* is proportional to *n^H^*

$$\begin{array}{ll} X_{i,n}\propto s^{-H}X_{i,sn}, & \text{(15)}\\ m_{n}\propto pn^{H},\;which\;yields\;\log\;m_{n}\propto \log p+H\log n, & \text{(16)} \end{array}$$

where *H* is the Hurst exponent. *H* ranges between 0 and 1. Increments *Y_i_* = *X_i_* − *X_i−_*_1_ of a non-stationary fBm signal yield a stationary fGn signal and vice versa, cumulative summation of an fGn signal results in an fBm signal. Note that most methods listed below that have been developed to analyze statistical fractal processes share the philosophy of Eq. 15 in that in their own ways all attempt to capture the power law scaling in the various statistical measures of the evaluated time series (Eke et al., 2002).

### Monofractal methods

Here we focus on widely used monofractal methods selected from those in the literature.

#### Time domain methods

##### Detrended fluctuation analysis

The method of Peng et al. (1994) begins with the signal summed and the mean subtracted

(17) $$Y_{j}=\overset{j}{\underset{i=1}{\sum }}X_{i}-\left\langle X\right\rangle .$$

Then the local trend *Y_j,n_* is estimated in non-overlapping windows of equal length *n*, using least-square fit on the data. For a given window size *n* the fluctuation is determined as the variance upon the local trend:

(18) $$F_{n}=\sqrt{\frac{1}{N}\overset{N}{\underset{j=1}{\sum }}{\left(Y_{j}-Y_{j,n}\right)}^{2},}$$

For fBm processes of length *N* with non-overlapping windows of size *n* the fluctuation depends on the window size *n* in a power law fashion:

(19) $$F_{n}\propto pn^{\alpha },\;\;\text{and}$$

$$\begin{array}{ll} \alpha =\underset{n\to 0}{\lim}\frac{\log F_{n}}{\log n}. & \text{(20)} \end{array}$$

If *X_i_* is an fGn signal then *Y_j_* will be an fBm signal. *F_n_* then is equivalent to *m_n_* of Eq. 16 yielding *F_n_*∝*pn^H^* therefore in this case α = *H*. If *X_i_* is an fBm signal then *Y_j_* will be a summed fBm signal. Then *F_n_*∝*pn^H + 1^*, where α = *H* + 1 (Peng et al., 1994).

##### Signal summation conversion method

This method was first introduced by Eke et al. (2000) for enhancing signal classification as a variant of the scaled windowed variance (SWV) analysis of Mandelbrot (1985) as further developed by Peng et al. (1994).

Fluctuations of a parameter over time can be characterized by calculating the standard deviation

(21) $$SD_{n}=\sqrt{\frac{1}{N-1}\overset{N}{\underset{i=1}{\sum }}{\left(X_{i}-\left\langle X\right\rangle \right)}^{2}.}$$

For fBm processes of length *N* when divided into non-overlapping windows of size *n* as Eq. 21 predicts the standard deviation within the window, *s_n_*, depends on the window size *n* in a power law fashion:

(22) $$SD_{n}\propto pn^{H},$$

and

(23) $$H=\underset{n\to 0}{\lim}\frac{\log SD_{n}}{\log n}.$$

In practice SD*_n_*’s calculated for each segment of length *n* of the time series are averaged for the signal at each window size. The standard method applies no trend correction. Trend in the signal seen within a given window can be corrected either by subtracting a linearly estimated trend (line detrended version) or the values of a line bridging the first and last values of the signal (bridge detrended version; Cannon et al., 1997). This method can only be applied to fBm signals or cumulatively summed fGn signals.

The signal summation conversion (SSC) method was first used for enhanced signal classification according to the dichotomous fGn/fBm model (Eke et al., 2000). There are two steps: (1) calculate from *X_i_* its cumulative sum (this converts an fGn to an fBm or converts an fBm to its cumulant), and (2) use the bdSWV method to calculate from the cumulant series ${Ĥ}^{\prime }$. The interpretation of ${Ĥ}^{\prime }$ is that when $0<{Ĥ}^{\prime }\leq 1,$ then *X_i_* is an fGn with ${Ĥ}^{\prime }$. Alternatively, when ${Ĥ}^{\prime }>1,$ then the cumulant series is identified as an fBm signal of $Ĥ={Ĥ}^{\prime }-1.$ As seen, in order to keep ${Ĥ}^{\prime }$ scaled within the [0,1] range, in the original version of the method in the fBm case 1 was subtracted from the estimate of *H*. Given that the SSC method handles fGn and fBm signals alike, we eliminate this step and report values as $0<{Ĥ}^{\prime }<1$ for fGn and $1<{Ĥ}^{\prime }<2$ for fBm signals referring ${Ĥ}^{\prime }$ as the “extended” Hurst exponent. This way, the mere value of the Hurst exponent would reflect on signal class, the focus of fractal time series analysis strategy. Also the use of ${Ĥ}^{\prime }$ would greatly facilitate reviewing the results of numerical performance analyses.

Real-time implementations of SSC and Detrended Fluctuation Analysis (DFA) methods have been recently reported (Hartmann et al., 2012).

#### Frequency domain method

Fractal analysis can also be done in the frequency domain using methods such as the power spectral density (PSD) analysis (Fougere, 1985; Weitkunat, 1991; Eke et al., 2000).

##### Power spectral density analysis (^low^PSD_w,e_)

A time series can be represented as a sum of cosine wave components of different frequencies:

(24) $$X_{i}=\overset{N∕2}{\underset{n=0}{\sum }}A_{n}\cos\left[\omega_{n}t_{i}+\varphi_{n}\right]=\overset{N∕2}{\underset{n=0}{\sum }}A_{n}\cos\left[\frac{2\pi n}{N}i+\varphi_{n}\right],$$

where *A_n_* is the amplitude and Φ*_n_* is the phase of the cosine-component with ω*_n_* angular frequency. The commonly used sample frequency is *f_n_* = ω*_n_*/2π. The *A_n_*(*f_n_*), Φ*_n_*(*f_n_*), and $A_{n}^{2}\left(f_{n}\right)$ functions are termed amplitude, phase, and power spectrum of the signal, respectively. These spectra can be determined by an effective computational technique, the fast Fourier transform (FFT). The power spectrum (periodogram, PSD) of a fractal process is a power law relationship

$$\begin{array}{llll} & A_{n}^{2}\propto p\omega_{n}^{-\beta },\;\text{or}{\left|A\left(f\right)\right|}^{2}\propto 1∕f^{\beta }\;\text{which yields}\;\beta = & & \\ & \underset{n\to 0}{\lim}\frac{\log A_{n}^{2}}{\log f_{n}}, & & \text{(25)} \end{array}$$

where β is termed spectral index. The power law relationship expresses the idea that as one doubles the frequency the power changes by the same fraction (2^−β^) regardless of the chosen frequency, i.e., the ratio is independent of where one is on the frequency scale.

The signal has to be preprocessed before applying the FFT (subtraction of mean, windowing, and endmatching, i.e., bridge detrending). Discarding the high power frequency estimates improves the precision of the estimates of β (Fougere, 1985; Eke et al., 2000). Eke et al. (2000) introduced this version denoted as ^low^PSD *_w,e_* as a fractal analytical tool.

#### Time-frequency domain method

*Fractal wavelet analysis* uses a waveform of limited duration with an average value of zero for variable-sized windowing allowing an equally precise characterization of low and high frequency dynamics in the signal. The wavelet analysis breaks up a signal into shifted and stretched versions of the original wavelet. In other words, instead of a time-frequency domain it rather uses a time-scale domain, which is extremely useful not only in monofractal but multifractal analysis, too. One such way to estimate *H* is by the averaged wavelet coefficient (AWC) method (Simonsen and Hansen, 1998). The most commonly used analyzing wavelet is the second derivative of a standard normalized Gaussian function, which is:

(26) $$\psi \left(t\right)=\frac{d^{2}}{dt^{2}}e^{-\frac{t^{2}}{2}}.$$

The scaled and translated version of the analyzing wavelet is given by

(27) $$\psi_{a;b}\left(t\right)=\psi \left(\frac{t-b}{a}\right),$$

where the scale parameter is *a*, and the translation parameter *b*.

The wavelet transformation is essentially a convolution operation in the time domain:

(28) $$W_{\psi }\left[X\right]\left(a,b\right)=\frac{1}{a}\overset{+\infty }{\underset{-\infty }{\int }}X\left(t\right)\cdot \psi_{a;b}dt.$$

From Eq. 16, one can easily derive how the self-affinity of an fBm signal *X*(*t*) determines its continuous wavelet transform (CWT) coefficients:

(29) $$W\left[X\right]\left(sa,sb\right){=}_{d}s^{\frac{1}{2}+H}W\left[X\right]\left(a,b\right).$$

The AWC method is based on Eq. 29 (Simonsen and Hansen, 1998) and can be applied to fBm signals or to cumulatively summed fGn signals.

### Multifractal methods

Three analysis methods are described here; all use different statistical moments (termed *q*-th order) of the selected measure to evaluate the signal’s multifractality. Despite of certain inherent drawbacks, these methods are widely used in the literature, and can obtain reliable results if their use is proper with limitations considered.

#### Time domain methods

Below, the Multifractal DFA (MF-DFA; Kantelhardt et al., 2002) and the recently published Multifractal Detrended Moving Average (MF-DMA; Gu and Zhou, 2010) will be reviewed. We will focus on MF-DMA, but since it is similar to MF-DFA, their differences will be pointed out, too. They rely on a measure of fluctuation, *F*, as in their monofractal variant (Peng et al., 1994), and differ in calculating the *q*-th order moments of the fluctuation function.

**Figure 4:**
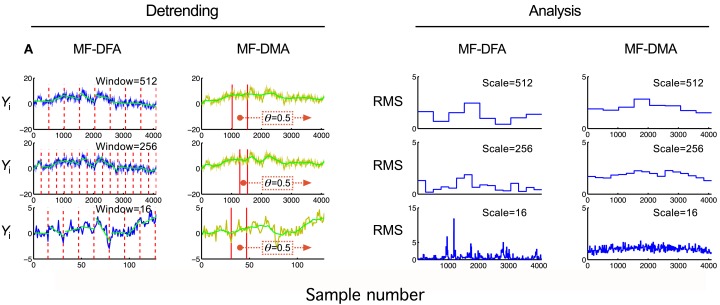
**Detrending scheme and fluctuation analysis for MF-DFA and MF-DMA methods**. The detrending strategy for MF-DFA **(A)** is that the signal is divided into a set of non-overlapping windows of different sizes, and a local low-order polynomial (typically linear) fit (shown in green) is removed from each window’s data. In contrast, MF-DMA **(B)** removes the moving average point-by-point calculated in different window sizes around the processed point with a position given by θ. This parameter describes the delay between the moving average function and the original signal. Its value is taken from [0, 1] interval, 0 meaning only from signal values on the left (“*backward*,” past), in contrast with 1 meaning that only signal values to the right (“*forward*,” future) are used for calculating ${Ỹ}_{j}.$ The centrally positioned sliding window corresponds to the case of θ = 0.5 balancing contributions from the past and the future to the reference point. The approaches of MF-DFA and MF-DMA thus ought to yield different detrended signals, whose calculated moments **(C,D)** and Eqs 33 and 34 obtained by the analysis should also be somewhat different.

*Step 1 – calculating signal profile, Y_j_, by cumulative summation*. It is essentially the same as in Eq. 17, however note that in DFA methods, the mean of the whole signal is subtracted before summation, while in DMA methods this is carried out locally in step 3.
*Step 2 – calculating the moving average function*,${Ỹ}_{j}.$
  (30) $${Ỹ}_{j}=\frac{1}{n}\cdot \overset{\left[\left(n-1\right)\left(1-\theta \right)\right]}{\underset{k=-\left[\left(n-1\right)\theta \right]}{\sum }}y_{t-k}$$
  For further details, see Figure 4.
*Step 3 – detrending by moving average:* By subtracting ${Ỹ}_{t}$ a residual signal, ε*_t_*, is obtained:
  (31) $$\varepsilon_{t}=Y_{t}-{Ỹ}_{t},$$
  where *n*−[(*n*−1) · θ] ≤ *t* ≤ *N*−[(*n*−1)· θ].
  This fundamental step of the DMA methods is essentially different from the detrending step of DFA methods (See Figure 4).
*Step 4 – calculation of fluctuation measure*. The signal is split into *N_n_* = [*N*/*n* − 1] number of windows (See Figure 4), ε(*v*), where *v* refers to the index of a given window. The fluctuating process is characterized by *F_v_*(*n*), which is given as a function of window size, *n*:
  (32) $$F_{v}^{2}\left(n\right)=\frac{1}{n}\cdot \overset{n}{\underset{t=1}{\sum }}\varepsilon_{t}^{2}\left(v\right).$$
*Step 5 – calculation of *q*-th order moments of the fluctuation function*.

(33) $$F_{q}\left(n\right)={\left(\frac{1}{N_{n}}\cdot \overset{N_{n}}{\underset{v=1}{\sum }}F_{v}^{q}\left(n\right)\right)}^{1∕q}.$$

For *q* = 2, the algorithm reduces to the monofractal DMA method. For the special case *q* = 0, *F_q_*(*n*) can be obtained as a limit value that can be expressed in a closed form:

(34) $$\log\left[F_{0}\left(n\right)\right]=\frac{1}{N_{n}}\cdot \overset{N_{n}}{\underset{v=1}{\sum }}\log\left[F_{v}\left(n\right)\right].$$

Relation of the *q*-th order moment of the fluctuation measure and *H*(*q*) follows a power law:

(35) $$F_{q}\left(n\right)\propto n^{H\left(q\right)}.$$

Thus *H*(*q*) can be estimated as the slope of the least-square fitted regression line between log *n* and log [*F_q_*(*n*)]. Finally, Mandelbrot spectrum is obtained with subsequent application of multifractal formalism equations (Eqs 9–12) yielding multifractal features τ(*q*), *D*(*h*).

#### Time-frequency domain methods

**Figure 5:**
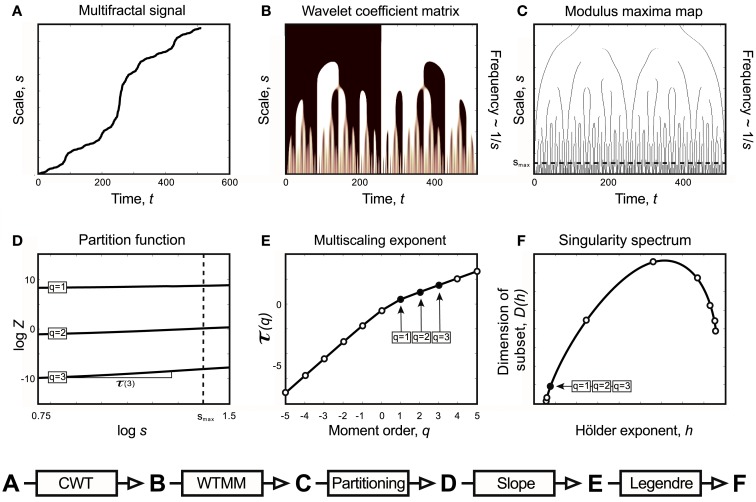
**Relations of Continuous Wavelet Transform operation, Wavelet Transform Modulus Maxima method, and multifractal formalism to obtain singularity spectrum of an ideal multifractal**. Devil’s staircase with weight factors *p*_1_ = *p*_3_ = 0.2, *p*_2_ = 0.6 was used to model an ideal multifractal time series **(A)**. The wavelet coefficient matrix **(B)** is obtained by continuous wavelet transform in the time-scale space. Modulus maxima map **(C)** containing the maxima lines across the scales defined by CWT. We call modulus maximum of the wavelet transform |W_ψ_[*X*](*t*, *s*_0_)|; any point (*t*_0_, *s*_0_), which corresponds to a local maximum of the modulus of |W_ψ_[*X*](*t*, *s*_0_)| is considered as a function of *t*. For a given scale, it means that |*W*_ψ_[*X*](*t*_0_, *s*_0_)| > |*W*_ψ_[*X*](*t*, *s*_0_)| for all *t* in the neighborhood right of *t*_0_, and |*W*_ψ_[*X*](*t*_0_, *s*_0_)| ≥ |*W*_ψ_[*X*](*t*, *s*_0_)| for all *t* in the neighborhood left of *t*_0_. Local maxima are chained, and in the subsequent calculations only maxima chains propagating to the finest scales are used (Mallat, 1999). Chaining local maxima is important, because it is proven that their distribution along multiple scales identifies and measures local singularities, which is tightly linked to the singularity spectrum. The moment-based partition function **(D)** separates singularities of various strength as coded in **(B,C)** as follows. *Z* is obtained for the range [*s*_min_, *s*] as the sum of moments of the wavelet coefficients belonging to those along a set of maxima lines at *s* [shown as circles in **(C)**]. This definition corresponds to a “scale-adapted” partition with wavelets at different sizes. A moment-based set of *Z* are plotted in a log-log representation as shown in **(D)**. Notice that these log *Z*(log *s*) functions are lines representing the power law behavior of the multifractal signal within the scaling range shown. Therefore when the slope of each and every log *Z*(log *s*) lines are plotted as a function of moment order, *q*, it yields τ(*q*) **(E)**. From τ(*q*) via Legendre transform the singularity spectrum, *D*(*h*) **(F)**, is obtained (See Chapter 2, Figure 3).

Wavelet analysis methods can be used to estimate the singularity spectrum of a multifractal signal by exploiting the multifractal formalism (Muzy et al., 1991, 1993, 1994; Mallat and Hwang, 1992; Bacry et al., 1993; Arneodo et al., 1995, 1998; Mallat, 1999; Figure 5). Wavelet transform modulus maxima (WTMM) has strong theoretical basis and has been widely used in natural sciences to assess multifractality.

*Step 1 – continuous wavelet transformation:* This step is essentially the same as described previously in Eqs 26–28 yielding a matrix of wavelet coefficients (Figure 5B):
  (36) $$W\equiv \left[w\left(i_{t},i_{s}\right)\right],$$
  where *w*(*i_t_*, *i_s_*) = |*W*_ψ_[*X*](*t*, *s*)|, *i*_s_ is the scaling index, where *s* = *s*_min_, …, *s*_max_ and *i_t_* = 1, 2, …, *N*, where *t* is the sampling time of each successive data point.
*Step 2 – chaining local maxima:* The term modulus maxima describes any point (*t*_0_, *s*_0_) where |*W*_ψ_=[*X*](*t*, *s*)| is a local maximum at *t* = *t*_0_:
  (37) $$\frac{\partial W_{\psi }\left[X\right]\left(t_{0},s_{0}\right)}{\partial t}=0.$$
  This local maximum is strict in terms of its relation to *t*_0_ in its immediate vicinity. These local maxima are to be chained by interconnection to form a local maxima line in the space-scale plane (*t*, *s*) (See Figure 5C).
*Step 3 – calculating partition function*. With the aid of partition function (Eq. 7, Figure 5D), singular behavior of the multifractal time series can be isolated. Wavelet coefficients along maxima chains are considered as μ measures.
  (38) $$Z\left(s,q\right)=\underset{\ell \in L\left(s\right)}{\sum }{\left|w\left(i_{s},i_{t}\right)\right|}^{q}.$$
  Summation is executed along maxima chains (ℓ), the set of all maxima lines is marked by *L*(*s*).
*Step 4 – calculating singularity spectra and parameters of multifractality*. The following step is to determine the multiscaling exponent, τ(*q*) by *H*(*q*), and then using Eqs 10–12 to give full quantification of the multifractal nature.

## Characterization of Methods

Before the application of fractal analysis methods, their behavior should be thoroughly evaluated on a large set of signals with known scale-free structure and broad representation (Bassingthwaighte and Raymond, 1994, 1995; Caccia et al., 1997; Cannon et al., 1997; Eke et al., 2000, 2002; Turiel et al., 2006). Signal classification, estimating performance in terms of precision and limitations of the methods should be clarified during characterization. The capability of multifractal analysis to distinguish between mono- and multifractal processes should also be evaluated.

Stationarity of a signal is an important property for pairing with a compatible fractal analysis tool (see Table 2 in Eke et al., 2002). In addition, all methods have some degree of inherent bias and variance in their estimates of the scaling exponent bearing great importance due to their influence on the results, which can be misinterpreted as a consequence of this effect. The goal of performance analysis is therefore to characterize the reliability of selected fractal tools in estimating fractal parameters on synthesized time series. This should be carried out at least for a range of signal sizes and structures similar to the empirical dataset, so that the reliability of fractal estimates could be accurately determined.

Extensive results obtained with our monofractal framework have been reported elsewhere (Eke et al., 2000, 2002), but for the sake of comparison it will be briefly described. Our multifractal testing framework is aimed to demonstrate relevant features of MF-DFA and MF-DMA method, utilizing the equations described in Section “Implementation of Fractal Time Series Analyses.”

### Testing framework for multifractal tools on monofractals

Monofractal signals of known autocorrelation (AC) structure can be synthesized based on their power law scaling. The method of Davies and Harte (1987) (DHM for short) produces an exact fGn signal using its special correlation structure, which is a consequence of the power law scaling of the related fBm signal in the *time domain* (Eq. 19). It is important, that different realizations can be generated with DHM at a given signal length and Hurst exponent, which consists of a statistical distribution of similarly structured and sized monofractals.

The next question is how to define meaningful end-points for the tests? For ideal monofractals with a given length and true *H*, Mean Square Error (MSE) is a good descriptor: it can be calculated for each set of series of known *H* and particular signal length, *N* (Eke et al., 2002). It carries a combined information about bias and variance, as MSE = bias^2^ + variance.

**Figure 6:**
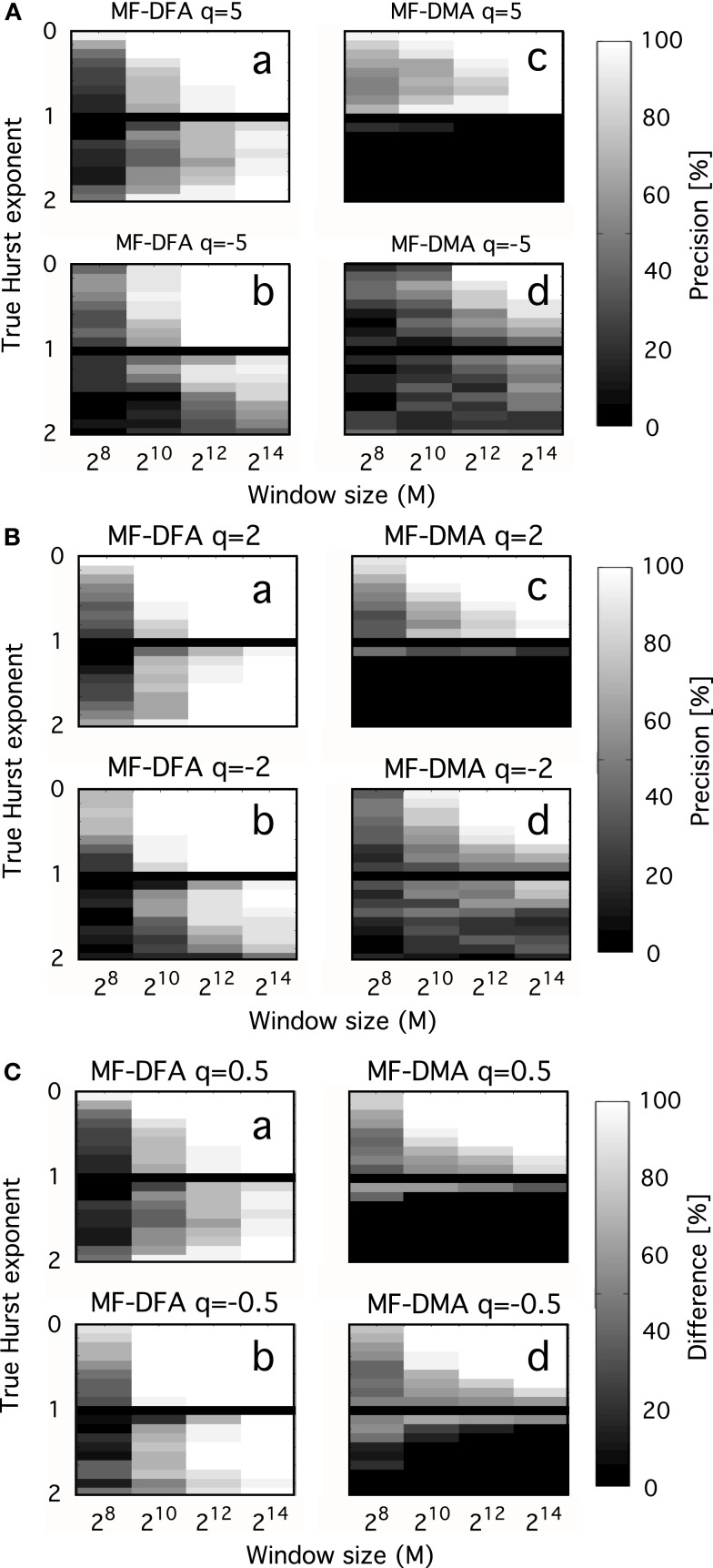
**Precision as a function of moment order, signal length, and Hurst exponent**. Precision of MF-DFA [left side of **(A–C)**] and MF-DMA [right side of **(A–C)**] as a function of *q*, *H*_true_, *N*. fGn and fBm signals were generated by DHM with length of 2^8^, 2^10^, 2^12^, 2^14^, and *H*_true_ increased from 0.1 to 1.9 in steps of 0.1, skipping *H*_true_ = 1 (corresponding to 1/*f* boundary seen as the black horizontal line in the middle). Estimation of the generalized Hurst exponent should not depend on *q*, as monofractal’s *H*(*q*) is a theoretically constant function scattering around *H*_true_ across different order of moments. The intensity-coded precision index is proportional to the number of estimates of *H* falling into the range of *H*_true_ ± 0.1, with lighter areas indicating more precise estimation. Calculation of this measure is based on 20 realizations for each *q*, *H*_true_, *N*. **(A)** Performance of methods for *q* = ± 5. **(B)** Performance of methods for *q* = ± 2. **(C)** Performance of methods for *q* = ± 0.5. Besides the clear dependence of precision on *H*_true_ and *N*, influence of moment order is also evident, given that the lightest areas corresponding to the most reliable estimates tend to increase in parallel with moment order approaching 0 [Note the trend from **(A–C)**]. The lower half of the plots indicates that MF-DFA is applicable for signals of both types, while MF-DMA is reliable only on fGn signals. This result is further supported by the paper of Gao et al. (2006), who demonstrated a saturation of DMA at 1 for *H* when the true extended Hurst exponent exceeds 1 (thus it is non-stationary)

Interpreting the multifaceted results of numerical experiments is a complex task. It can be facilitated if they are plotted in a properly selected set of independent variable with impact shown in intensity-coded representations (Figure 6; Eke et al., 2002). Precision index is determined as the ratio of results falling in the interval of [*H*_true_ – *H*_dev_, *H*_true_ + *H*_dev_], where *H*_dev_ is an arbitrarily chosen value referring to the tolerable degree of deviation.

In the monofractal testing framework, we used DHM-signals to evaluate the performance of MF-DMA (Gu and Zhou, 2010) and MF-DFA (Gu and Zhou, 2006), by the code obtained from http://rce.ecust.edu.cn/index.php/en/research/129-multifractalanalysis. It was implemented in Matlab, in accordance with Eqs 17 and 30–35. As seen in Figure 6, precision of MF-DFA and MF-DMA depends on *N*, *H*, and the order of moment.

**Figure 7:**
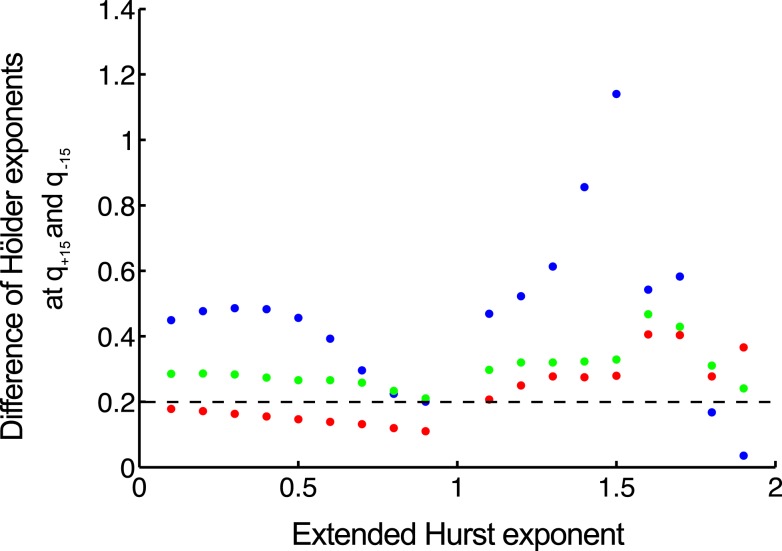
**Separating monofractals from multifractals**. Δ*h* values obtained by MF-DFA (as difference of Hölder exponents at *q* = + 15 and *q* = −15) are shown for monofractals with length of 2^10^ (blue), 2^12^ (green), 2^14^ (red). It is clearly shown that longer signals are characterized by lower Δ*h*, and its value below 0.2 means that true multifractality is unlikely present (Grech and Pamula, 2012). Signals were created by DHM at extended Hurst exponents of 0–1.9 with a step of 0.1.

In order to compare the methods in distinguishing multifractality, end-points should be defined reflecting the narrow or wide distribution of Hölder exponents. We select a valid endpoint Δ*h* proposed by Grech and Pamula (2012), which is the difference of Hölder exponents corresponding to *q* = −15 and *q* = + 15 (Figure 7).

### Testing approaches for multifractal tools on multifractals

Extending the dichotomous model of fGn/fBm signals (introduced in context of monofractals; Mandelbrot and Ness, 1968; Eke et al., 2000) toward multifractal time series is reasonable as it can account for essential features of natural processes exhibiting local power law scaling. Description of an algorithm creating multifractional Brownian motion (mBm) and multifractional Gaussian noise (mGn) can be found here (Hosking, 1984), while implementation of such code can be found on the net (URL1: http://fraclab.saclay.inria.fr/, URL2: www.ntnu.edu/inm/geri/software). Given that these algorithms require Hölder trajectories as inputs, multifractality cannot be defined exactly on a finite set, which is a common problem of such synthesis methods. Selecting a set of meaningful trajectories is a challenging task: it should resemble those of empirical processes and meet the analytical criteria of the selected algorithms (such criteria are mentioned in Concept of Fractal Time Series Analyses).

On the contrary, iterative cascades defined with analytic functions are not influenced by the perplexity of definitions associated with multifractality outlined in the previous paragraph, given that their value at every real point of the theoretical singularity spectrum is known. Due to their simplicity, binomial cascades (Kantelhardt et al., 2002; Makowiec et al., 2012) and Devil’s staircases (Mandelbrot, 1983; Faghfouri and Kinsner, 2005) are common examples of theoretical multifractals used for testing purposes. A major drawback of this approach is that these mathematical objects do not account for features in empirical datasets, but can still be useful in comparing reported results.

The most extensive test of multifractal algorithms which used a testing framework of signals synthesized according to the model introduced by Benzi et al. (1993) was reported by Turiel et al. (2006). Briefly, it is a wavelet-based method for constructing a signal with predefined properties of multifractal structuring with explicit relation to its singularity spectrum. Since the latter can be manipulated, the features of the resulting multifractal signal could be better controlled. The philosophy of this approach is very similar to that of Davies and Harte (1987) in that a family of multifractal signals of identical singularity spectra can be generated by incorporating predefined distributions (log-Poisson or log-Normal) giving rise to controlled variability of realizations. Additionally, using log-Poisson distribution would yield multifractals with a bounded set of Hölder exponents in that being similar to those of empirical multifractals. To conclude, this testing framework should merit further investigation.

## Analytical Strategy

**Figure 8:**
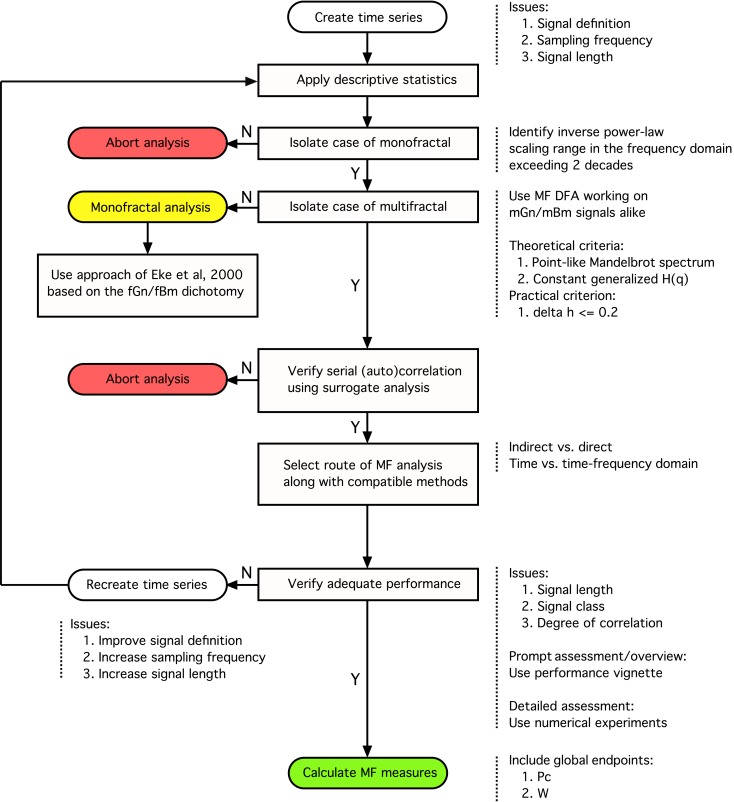
**Analytical strategy for fractal time series analysis**. Toward obtaining a reliable (multi)fractal parameter, which is the purpose of the analysis, the first step to take is to collect a high definition dataset representing the temporal signal, *X*(*t*), ensuring adequate definition. Provided that quality-controlled, adequate length of signal, *X_i_*, was acquired at a sufficient frequency sampling *X*(*t*) (Eke et al., 2002), scale-free processes can be characterized in terms of either a single global or a distribution of many local scaling exponents, the former pertinent to a monofractal, the latter to a multifractal signal, respectively (Figure 1). A detailed flowchart of our monofractal analytical strategy has been reported earlier (Eke et al., 2000, 2002), hence only some of its introductory elements are incorporated here. The signal-to-noise ratio – as part of signal definition – is a source of concern in preprocessing the signal. Ensuring the domination of the underlying physiological processes over inherent noise is a critical issue, which – if not dealt with properly – will have a detrimental effect on the correlation structure of the signal. Endogenous filtering algorithms of the manufacturers of MRI scanners could be operating in potentially relevant frequency ranges of fractal analysis aimed at trend or noise removal (Jezzard and Song, 1996). In case of BOLD signals, this problem may prove hard to track as the system noise may cause a temporally (i.e., serially) correlated error in the measurement (Zarahn et al., 1997). This may alter the autocorrelation structure of the signal with embedded physiological content (Herman et al., 2011). Various aspects of temporal smoothing have been discussed in Friston et al. (2000). To conclude, scale-free properties of the signal must be preserved during steps carried out before fractal analysis, otherwise the physiologically relevant internal structuring of the BOLD signal cannot possibly be revealed (Herman et al., 2011). Once a multifractal has been isolated by a class-independent method, such as MF-DFA, we can only assume that the multifractal structuring of the signal is due to serial correlation. As autocorrelation structure of the signal can reflect a broad probability distribution, surrogate analysis is needed on a shuffled signal – which destroys this correlation – to ensure that the origin of the scale-invariance is due to genuine autocorrelation in the signal (Kantelhardt:2002]). The null-hypothesis (the signal is not multifractal) is rejected if multifractal measures determined for the raw and surrogate sets are different. This procedure is similar to verifying the presence of deterministic chaos (Herman:2006]). Attention should be given to select the scaling range properly: involving the finest and coarsest scales in calculating *H*(*q*) would greatly impair its estimate. The range of moments should be selected such that sufficient range of singularity spectrum is revealed, allowing for the calculation of scalar multifractal descriptors such as *P*_c_. Next, one has to decide as to which path of the detailed multifractal analysis to choose (indirect vs. direct or time vs. time-frequency domain)? Each of these paths would have advantageous and disadvantageous contributions to the final results to consider. The methods of analysis must be selected compatible to the path taken. Once methods have been chosen, their performance (precision) ought to be evaluated. With adequate performance verified, the multifractal analyses can then be followed by attempts to find physiological correlates for the estimates of (multi)fractal parameters.

In this article we expand our previously published monofractal analytical strategy to incorporate some fundamental issues associated with multifractal analyses keeping how these can be applied to BOLD time series in focus. Progress along the steps of the perplexed fractal analysis should be guided by a consolidated – preferably model-based– view on the issues involved (See Figure 8).

**Figure 9:**
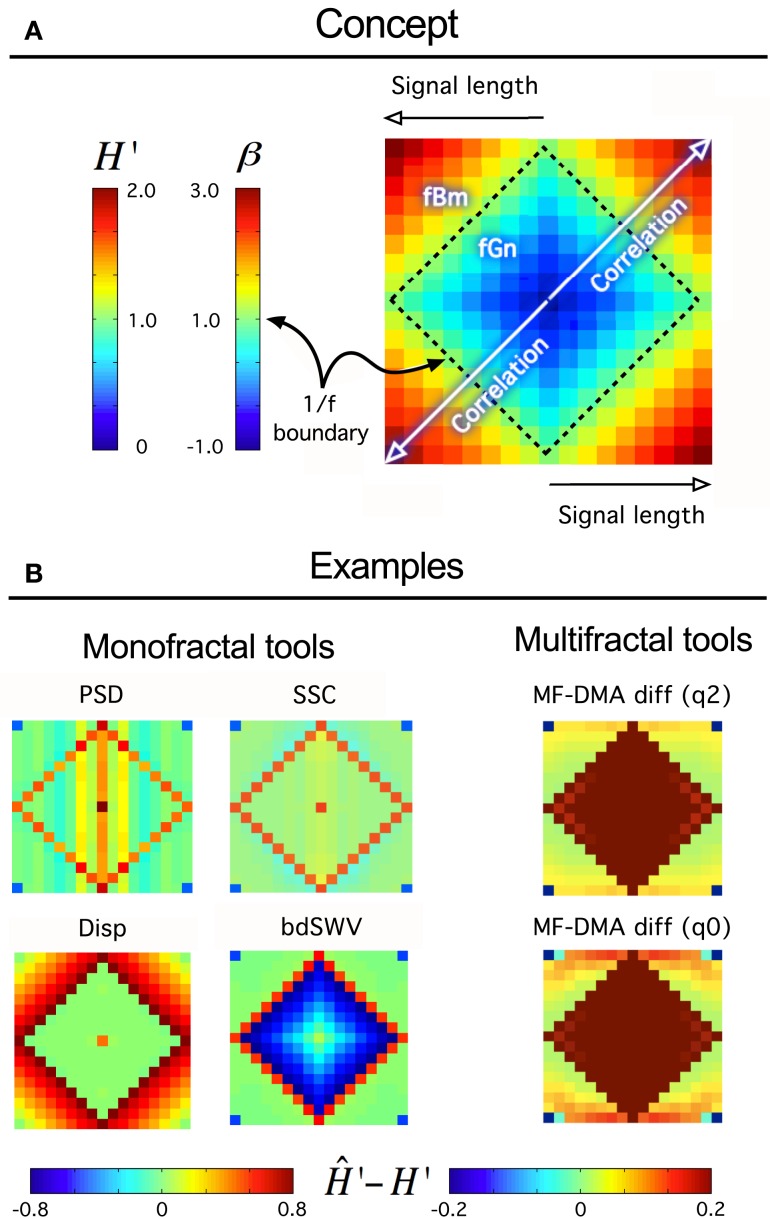
**Fractal tool performance vignette**. It provides a quick assessment of any fractal time series tool’s performance. As such can be useful as a method of standardization and/or comparison of various algorithms. Technically, a vignette is created as any given fractal time series method evaluates a volume of synthesized time series for a particular fractal parameter. The results are converted to extended *H*′ as *H*′ = *H*_fGn_, *H*′ = *H*_fBm_ + 1 using a conversion table between *H* and other fractal parameters (Eke et al., 2002). The signals are generated for a range of length, *L* [*L*_min_, *L*_max_] in increments of Δ*L*, and for the full range of the fGn/fBm dichotomy at β or *H*′ at given increments of the exponent, Δ*H’* by the DHM method (Davies and Harte, 1987; Eke et al., 2000). The volume is created from these signals arranged in a square raster, which will correspond to one of four identical quadrants of the vignette. Once the analysis by a fractal tool has been carried out the results are plotted in a square array as shown in **(A)** such a way that fGn signals occupy a square created by the four identical quadrants. The 1/*f* boundary separating the fGn from the fBm range can be easily identified as plotted with a midscale color. Warmer colors indicate over-, cooler colors underestimation of the scaling exponent at the particular signal length or degree of correlation. When applied to class-independent or dependent methods **(B)**, like PSD, SSC (**B**, upper half) or Disp (dispersional analysis) and bdSWV (bridge detrended Scaled Window Variance) (**B**, lower half), respectively, an immediate conclusion on signal performance can be drawn: PSD and SSC can be used for fGn and fBm signals alike (except in the vicinity of the 1/*f* boundary) and SSC is more precise. Disp (Bassingthwaighte and Raymond, 1995; Eke et al., 2000, 2002) and bdSWV (Eke et al., 2000, 2002), two class-dependent methods of excellent performance (note the midscale colored area in the fGn and fBm domains, respectively) do show up accordingly. The vignette is applicable to indicate the performance of multifractal methods, too. The monofractal *H* can be determined in two ways: in case of *q* = 2 from τ(*q*), and in case of *q* = 0 from *h*_max_ in the singularity spectrum.

A fundamental question should be answered whether it is worthy at all to take on the demanding task of fractal analysis? This can only be answered if one characterizes the signal in details according to the guideline shown in Figure 8 using tools of descriptive statistics and careful testing; first for the presence of monofractal and later that of multifractal scale-free features. At this end, we present here a new tool for an instantaneous and easy-to-do performance analysis (called “performance vignette”), which can facilitate this process and does not require special knowledge needed to carry out detailed numerical experiments on synthesized signals (Figure 9). The latter, however, cannot be omitted when full documentation of any particular fractal tool’s performance is needed. In that the vignette has been designed for prompt selection, overview, and comparison of various methods; not for their detailed analysis.

We sustain our recommendation that proper class-dependent or class-independent methods should be chosen.

We feel, that calculating global measures of multifractal scaling, such as *P*_c_ (Shimizu et al., 2004) or *W* (Wink et al., 2008), can help consolidating experimental findings in large fMRI BOLD volumes across many subjects and experimental paradigms. Based on our tests, we conclude that straightforward recommendations for multifractal analysis for the purpose of fMRI BOLD time series analysis needs further investigations.

## Pitfalls

### Sources of error

#### Problems emerging from inadequate signal definition (measurement sensitivity, length, sampling frequency)

##### Measurement sensitivity

The precondition of a reliable fMRI time series analysis is that the BOLD signal has adequate definition in terms of being a true-to-life representation of the underlying biology it samples. In particular, the fMRI BOLD measurement is aimed at detecting the contrast around blood filled compartments in magnetic susceptibility of blood and the surrounding medium in a uniform high field (Ogawa and Lee, 1990). A contrast develops from tissue water relaxation rate being affected by the paramagnetic vs. diamagnetic state of hemoglobin. The contrast increases with decreasing oxygenation of blood, a feature that renders the technique capable of detecting the combined effect of neuronal metabolism coupled via hemodynamics throughout the brain (Smith et al., 2002). As Ogawa and Lee (1990) demonstrated, the BOLD contrast increases with the strength of the main magnetic field, *B*_0_ (i.e., due to the sensitivity of the relaxation rate).

In his early paper (Lauterbur, 1973), Lauterbur gave clear evidence of the fact that resolution of magnetic resonance signals will strongly depend on *B*_0_. Newer generations of scanners with continuously improved performance were constructed utilizing this relation by incorporating magnets of increased strength (in case of human scanners from, i.e., 1.5–7T, in small animal scanners due to the smaller brain size with strength in the 4–17.2T range). Bullmore et al. (2001) showed indeed, that the performance of some statistical method and their results depended on the magnetic field used (1.5 vs. 3T); calling for caution and continuous reevaluation the methods in the given MRI settings.

**Figure 10:**
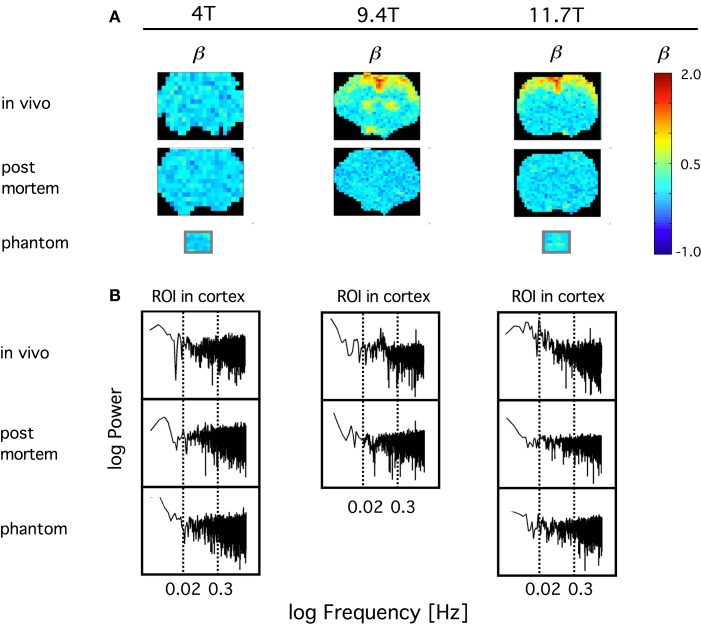
**Definition of spontaneous BOLD fluctuations critically depends on main field strength**. Exemplary coronal scans are shown obtained in anesthetized rat in MR scanner applying 4, 9.4, and 11.7T main external field. All fMRI data were collected at 5 Hz in length of 4096 (2^12^) images with gradient echo planar imaging (EPI) sequence using ^1^H surface coils (Hyder et al., 1995). **(A)** shows *in vivo* and *post mortem* maps of spectral index, β. β was calculated from the spectra of the voxel-wise BOLD time series by the PSD method for a restricted range of fluctuation frequency (0.02–0.3 Hz) found to exhibit inverse power law relationship [fractality; indicated by vertical dashed lines on the PSD plots in **(B)**]. In order to achieve a suitable contrast for the topology, β are color coded within the fGn range (from 0 to 1). Hence voxel data with β >  1 indicating the presence of fBm type fluctuations are displayed saturated (in red). β maps for water phantoms placed in the isocenter are also shown for comparison. Note, that the fractal pattern of internal structuring of the spontaneous BOLD signal cannot be captured at adequate definition at 4T as opposed to 11.7T, where the rate of scale-free rise of power toward low frequencies are thus the highest at about the same region of interest (ROI) located in the brain cortex. This dependence translates into an articulate *in vivo* topology with increasing *B*_0_. Also note that *in vivo* 4T cannot yield a clear topology of β when compared to *post mortem*, and that the well defined topology achieved at higher fields vanished *post mortem* indicating the link between β and the underlying physiology.

In order to confirm the impact of *B*_0_ on the sensitivity on the definition of the BOLD signal fluctuations, we have compared the spectral index (ß) of resting-state BOLD fluctuations *in vivo* to those *post mortem* and in a phantom in 4, 9.4, and 11.7T in anesthetized rats (Figure 10). What we have learned from this study was that in contrast with amplitude-wise optical measurements of cerebral oxygenation and hemodynamics such as near infrared spectroscopy (Eke et al., 2006), due to the contrast-detecting foundations of fMRI, signal definition cannot be characterized by comparing fluctuation ranges *in vivo* vs. *post mortem*. After death deoxyhemoglobin molecules are still present in the MRI voxels post-sacrifice and thus generate susceptibility-induced magnetic field gradients that would impact diffusion of tissue water molecules (Herman et al., 2011), a process that can generate fluctuating BOLD contrast without ongoing physiology. What matters is that *in vivo* the blood gets oxygenated and via the combined impact of neuronal metabolism, blood flow, and blood volume, the internal structuring of the BOLD contrast signal will change from close to random to a more correlated level as indicated by β, which is *in vivo* significantly higher than *post mortem*. Increasing field strength enhances this effect and yields a more articulated topology of β throughout the brain. Conversely, low field measurements favor the dominance of instrument noise in addition to being less sensitive in detecting the BOLD contrast. The inference of these preliminary data is that, given the BOLD contrast (and presumably even the spatial resolution) of our animal imaging, a 1.5T human scanner may not be of sufficient sensitivity to detect BOLD fluctuations at adequate definition for a reliable monofractal analysis, not to mention multifractal analysis known to require a much higher signal definition for an optimal performance that can be achieved in higher field scanners (Ciuciu et al., 2012).

While the use of fMRI is typically qualitative where the baseline is conveniently differenced away to reveal focal area(s) of interest (Shulman et al., 2007), this practice would not interfere with fractal time series analysis, given that scaling exponent is invariant to mean subtraction.

##### Length and sampling frequency

A signal is a sampled presentation of the underlying process, which generates it. Hence the sampling frequency must influence the extent the signal captures the true dynamics of the process, which is in the focus of fractal analysis irrespective if its analyzed in the time (in form of fluctuations) or in the frequency domain (in form of power distribution across the frequency scale). The sampling frequency should preferably be selected at least a magnitude higher than the highest frequency of the observed dynamics we would aim to capture.

The relationship between length and frequency can best be overviewed in the frequency domain along with the frequency components and aliasing artifact of the spectrum as seen in Figure 12 of Eke et al. (2002). Note, that the dynamics of interest can be best captured hence analyzed if the signal length is long; the sampling frequency is high, because it will provide a spectrum of many components with a weak artifactual impact of aliasing. Herman et al. (2011) have recently demonstrated this relationship on resting-state BOLD time series and concluded that lower frequency dynamics are better sampled by longer BOLD signals, whereas a high sampling rate is needed to capture dynamics in a wide bandwidth signal (See Figure 3 in Herman et al., 2011). In other words, inadequately low frequency is more detrimental to the result of fractal analysis than somewhat truncated signal.

Due to the discrete representation within the bounded temporal resolution of the signal, the precision of its fractal analysis increases with its length as demonstrated on simulated signals of known (true) fractal measures by the bias and variance of its estimates. The minimum length at which reasonable results can be expected depends not only on signal length but on the method of analysis and the degree of long-range correlation in the signal (as characterized by its *H*); an issue that has been explored in details for monofractal time series by the groups of Bassingthwaighte and Raymond (1994, 1995); Eke et al. (2000, 2002); Delignieres et al. (2006), and for multifractal methods by Turiel et al. (2006).

Multifractal analysis can be considered as an extension of monofractal analysis, which is explicitly true for moment-based methods: while in case of monofractals a scale-free measure is obtained at *q* = 2, the procedure for multifractals uses a set of different *q*-order moments. Think of *q* as a magnifier glass: different details of the investigated scale-free structure can be revealed at different magnification. However, if signal definition is poor due to short length or small sampling frequency, estimates of *D*(*h*) will become imprecise at large ±*q* (Figure 6). Since the order of *q* needed to obtain characteristic points of the singularity spectrum usually falls beyond *q* = ± 2, a longer time series is required to guarantee the needed resolution in this range. Hence, dependence of precision on signal length in case of multifractals is a more complicated issue, where the effect of spectral characteristics interacts with that of signal length (Turiel et al., 2006).

A reasonable conclusion is that the recommended minimum length for a reliable multifractal analysis ought to be longer than that found earlier for monofractal series (Eke et al., 2002; Delignieres et al., 2006).

#### Problem of signal class (fGn vs. fBm)

In fractal analysis, signal classification is a central issue (Eke et al., 2000) and should be regarded as a mandatory step when a tool is to be chosen from the class-dependent group. Living with the relative convenience of using a class-independent method does not render signal classification unnecessary given the great importance of proper interpretation of the findings that can be enhanced by knowing signal class.

**Figure 11:**
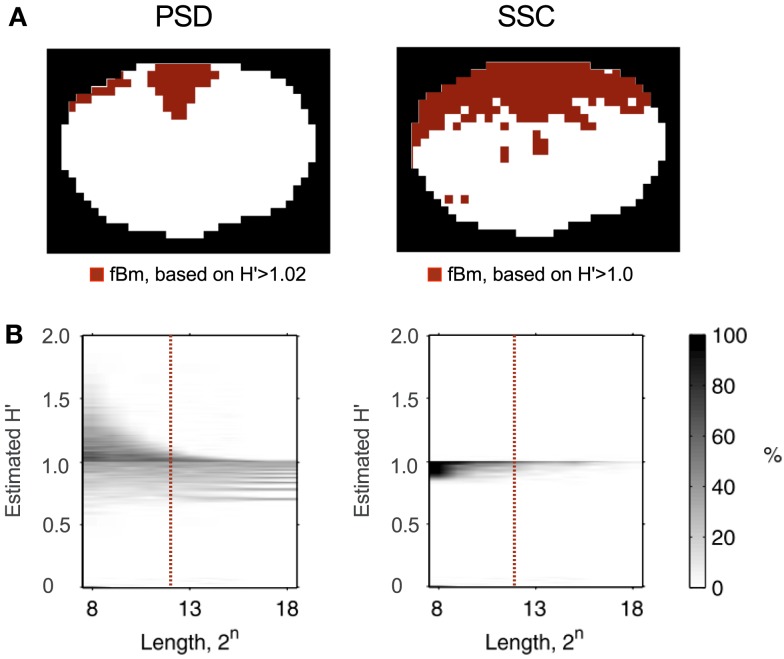
**Classifying rat fMRI BOLD data**. Signal classification was performed on the 11.7T BOLD dataset shown in Figure 10 by the PSD and SSC methods **(A)** previous tested in this capacity by Eke et al. (2002); misclassification rates for PSD and SSC are shown in the plots of **(B)** the lower panel. Because SSC is a much better classification tool, than PSD is, the classification topology will be drastically different for these two methods. The ROI’s corresponding to voxel-wise signals identified by SSC as non-stationary indeed do clearly delineate the anatomical boundaries of the brain cortex, while those by the PSD only the spots of highest β.

**Figure 12:**
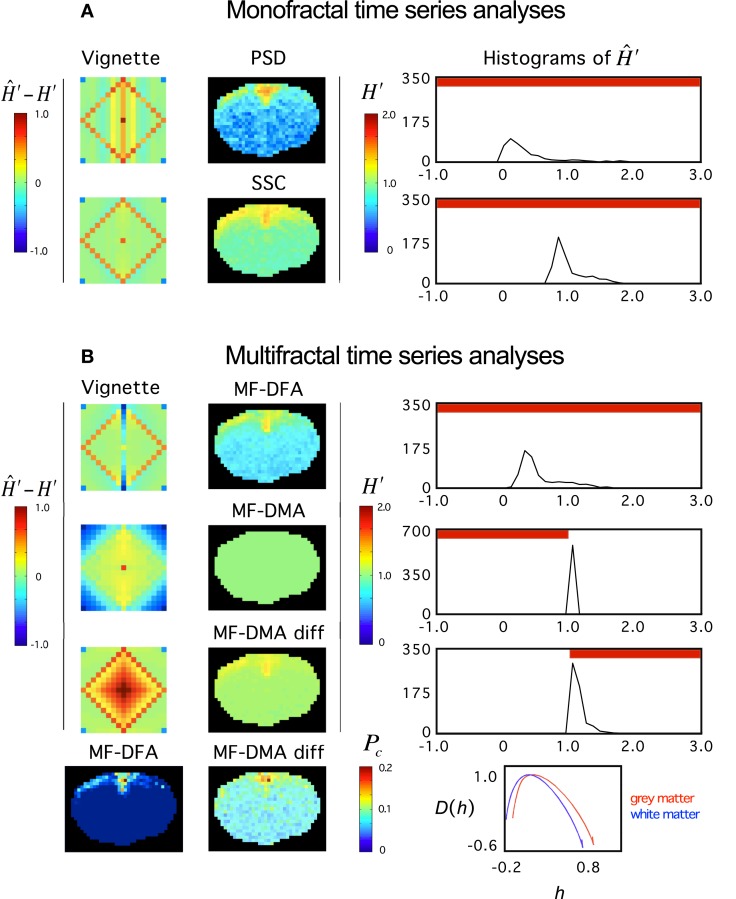
**Fractal analyses of rat fMRI BOLD data**. The 11.7T BOLD dataset shown in Figure 10 was analyzed monofractally **(A)** in the frequency domain by PSD, in the time domain by SSC, and multifractally **(B)** in the time domain by MF-DFA and MF-DMA methods. Estimates of spectral index were converted to extended Hurst exponent, *H*′. Our tool performance vignette is displayed next to the methods. Histograms of *H*′ computed from the fractal image data by SSC are shown. The vignette data reconfirms that SSC is superior over PSD as a monofractal tool. Due to the downward bias of PSD in the anticorrelated fGn range, *H*′ are significantly underestimated. Because SSC’s estimates are unbiased, the SSC topology should be considered realistic, which translates into a right shift of the SSC *H*′ histogram relative to that of PSD’s. Based on the vignette pattern, among the multifractal tools, MF-DFA works quite well on fGn and fBm signals, alike, while MF-DMA with fair performance in the fGn range but closer to the 1/*f* boundary, and fails on fBm signals of the set. For reasons mentioned above, the estimates of SSC should be taken as precise. Given that most values in the fGn range fall into the range of complete uncertainty of the MF-DMA (See Figure 6 at *q* = 2) and that MF-DMA cannot handle fBm signals, all estimates ends up being 1.0. Differencing the signals (including those of the vignette) changed the situation dramatically. As seen on the vignette, the originally fBm signals would be mapped into the fGn range that can be handled by MF-DMA very well. Actually better than the original fGn signals where slight overestimation is seen. This kind of behavior of MF-DMA may have inference with the findings of Gao et al. (2006). Also note, that the double differenced fGn signals end up being overestimated. These effects are worth to investigate in order to characterize the impact of the fGn/fBm dichotomy on the performance of these time domain multifractal tools when signals are being converted between the two classes. *P*_c_ – as a global multifractal measure – captures a topology similarly to the monofractal estimates. The corresponding singularity spectra do separate with the likelihood that the underlying multifractalities indeed differ.

Recently, Herman et al. (2011) found in the rat brain using monofractal analysis (PSD) that a significant population of fMRI BOLD signal fell into the non-stationary range of β. These non-stationary signals potentially interfere with resting-state connectivity studies using spatio-temporal volumes of fMRI BOLD. It is even more so, if SSC is used for signal classification (Figure 11) and analysis (Figure 12) shifting the histogram of *H*′ to the right.

For multifractals the problem and proposed solution is generally the same, but the impact of the fGn/fBm dichotomy on the multifractal measures is not a trivial issue. Our preliminary results reported here (Figure 12) are steps in this direction, but this issue calls for continuing efforts in the future. It seems that at least stationarity vs. non-stationarity is a valuable piece of information for selecting a concise model of multifractals.

#### Distinguishing monofractals from multifractals

Multifractal analysis of an exact monofractal rendered at ideal resolution (in infinite length, sampled at infinite frequency, at infinite sensitivity of detection) would yield a constant *H*(*q*), a linear dependence of τ on *q* and a point-like Mandelbrot spectrum with its Hölder exponent (*h*_max_) equal with its Hurst exponent.

Due to the finite and discrete nature of the signal, the singular behavior of a suspected scale-free process cannot be quantified perfectly. As a consequence, the homogeneity of a monofractal’s singularities cannot be captured by a multifractal analysis. The reason being is that due to numerical background noise (Grech and Pamula, 2012) – resulting from factors mentioned above – it would always smear the point-like singularity spectrum into one mimicking that of a multifractal. This is confirmed by the apparent uncertainties associated with the estimates of *H*(*q*) obtained at various moments in our simulations. All in all, multifractal analyses have been conceived in a manner that tends to view a monofractal as a multifractal.

In order to avoid false interpretation of the data, time series should be produced at the highest possible definition to ameliorate this effect and criteria should also be set up to distinguish the two entities in the signal to be analyzed. Numerical simulation has been demonstrated as a useful tool to work out a parameter that can be used to substantiate a monofractal/multifractal classification (Grech and Pamula, 2012; Figure 6).

#### Trends and noises

Empirical time series are typically non-linear, non-stationary and can be contaminated by noise and other signal components foreign to the fractal analysis of the system under observation. Trend is deterministic in its character and of typically low frequency in contrast with noise, which has a completely random structuring in a higher frequency range. Monofractal analysis methods are quite robust with respect of noise, thus in case of monofractals do not require preprocessing (Bassingthwaighte and Raymond, 1995). When uncorrelated noise is added to a multifractal process, the shape of its singularity spectrum will also be preserved (Figliola et al., 2010). However with correlated noise present, – known to impact fMRI BOLD time series – preprocessing should be considered (Friston et al., 2000), and if carried out, it should be done with an appropriate adaptive filter (Gao et al., 2010, 2011; Tung et al., 2011).

In case of wavelet-based methods, a polynomial trend can be removed based on the analyzing wavelet’s properties. However, if the trend has a different character (i.e., trigonometric or exponential), or it has more vanishing moments than that of the analyzing wavelet, the estimation of singularity spectrum will be impaired (See theorem 6.10 in Mallat’s book; Mallat, 1999).

Various detrending schemes have been developed to enhance performance of fluctuation analysis (FA) on detrended signals, which has been compared (Bashan et al., 2008). The most common trend removal is based on fitting a low-degree polynomial to local segments of the signal such as employed in DFA (Figure 4). In particular, DFA’s trend removal is credited for being very effective, however – as recently reported (Bryce and Sprague, 2012) – it can become inadequate if the trend ends up having a character different from the coded algorithm, which scenario cannot at all be excluded. A further problem is that the signal arbitrarily divided into analyzing window of different sizes in which trend removal is carried out based on *a priori* assumption (e.g., polynomial). This problem is exaggerated as by using partitioning of the signal into a set of non-overlapping windows and performing detrending in a window-based manner would not guarantee that the trend in each and every window would be identical with the assumed one. This is especially true for small windows, where trend tends to deviate from that in larger windows. Contrary to expectations, this critical finite size effect is always present, thus this pitfall can only be avoided if explicit detrending is applied by using adaptive methods (Gao et al., 2011).

To conclude, the recently reported uncontrollable bias to the results of DFA (Bryce and Sprague, 2012) raised major concern as to the reliability of FA with this detrending scheme. Thus if DFA is to be used, it should be done with special care taken in the application of more adaptive detrending analyses.

Finally, empirical mode decomposition (EMD) is a promising adaptive approach, one of whose feature is the ability to estimate trend explicitly. It also creates an opportunity to combine EMD with other fractal analysis methods like those based on FA to achieve a more reliable scale-free method (Qian et al., 2011).

#### Problems of moment-based methods

Using moment-based methods to estimate the Mandelbrot spectrum is a common approach with some drawbacks. Due to the discretized nature of the signal under analysis, small fluctuations cannot be resolved perfectly and therefore the Hölder exponents become biased in the range of their large negative moments (corresponding to the right tail of the singularity spectrum; Turiel et al., 2006). All moment-based methods are influenced by the linearization of the right tail thus yielding biased estimates of the negative statistical moments of the measure, μ (Turiel et al., 2006). This type of error cannot be eliminated with increasing the signal’s length (Turiel et al., 2006). In case of large fluctuations in the signal, numerical limitations become problematic when calculating large positive moments.

Problems associated with moment-based methods can be summarized as follows. Firstly, a carefully selected set of different order (*q*) statistical moments of μ should be calculated. Selecting too large negative and positive moments would lead to imprecise generalized Hurst exponent [*H*(*q*)] or multiscaling exponent (Figure 6; Ihlen, 2012). A sufficient range of *q* is needed, however, in order to characterize the global singular behavior of the studied time series. This is especially important in the evaluation of the spectrum, but from a practical point of view, the spectrum width at half maximum is sufficient to obtain *P*_c_, or *W* + /*W*−, that are frequently used lumped parameters in describing multifractal fMRI BOLD signals, too (Shimizu et al., 2004; Wink et al., 2008). In summary, precise estimation of singularity strength is needed at characteristic points of the spectrum: around its maximum (i.e., at *q* ≈ 0) and at its half maximum a dense definition is recommended. Thus, the optimal selection depends on the signal character and needs to be analyzed with several sets of *q*. In general, estimating spectrum between *q* = −5 and *q* = 5 is sufficient in biomedical applications, as proposed by Lashermes and Abry (2004). Secondly, methods implementing direct estimation of singularity spectra can be applied (Figure 3). One typical example is the gradient modulus wavelet projection (GMWP) method, which turned out to be superior to all other tested methods (WTMM, too) in terms of precision as reported by Turiel et al. (2006). It was shown that direct approaches can give quite good results in spite of the numerical challenges imposed by calculating the Hölder exponents (*h*) locally and without the need of using statistical moments and Legendre transform (Turiel et al., 2006). Strategies including the latter two approaches are widely used and can be considered reasonably, but not exclusively reliable in terms of their handling the numerical difficulties associated with multifractal analysis.

#### Problems of wavelet transform modulus maxima methods

In case of monofractals, the average wavelet coefficient method is the most effective and the easiest to implement (Simonsen and Hansen, 1998; Eke et al., 2002). It can be used for fBm and cumulatively summed fGn signals.

There are other issues related to this method, whose nature can be numerical on the one hand and theoretical on the other. For example, the first and last points of the signal exhibits artifactual scaling, improperly selected scales would impair the results considerably, etc. A well-selected analyzing wavelet also ensures reliable results, which is also proven for certain indirectly calculated partition functions (via Boltzmann weights; Kestener and Arneodo, 2003). The effect of the modifications addressing these issues is discussed in Faghfouri and Kinsner (2005) and a detailed test of WTMM is reported by Turiel et al. (2006).

Due to the difficulties in the reliable application of WTMM, other methods were developed in the field, the most promising one being the Wavelet Leader method (Lashermes et al., 2005; Serrano and Figliola, 2009), which has recently been applied to human fMRI BOLD signals (Ciuciu et al., 2012). As refinements of WTMM, the wavelet leader is beyond the scope of this review, the reader is referred to the cited references.

#### Identifying the spectral extent of monofractality within a signal

Verifying the presence of self-similarity, as one of the fundamental properties of monofractals is a key element of the analytical strategy of fractal time series analysis (Eke et al., 2000; Figure 8). It should be present within a sufficiently wide scaling range. In case of exact (mathematical) fractals the scaling range is unbounded. In natural fractal time series however it is typically restricted to a set of continuous temporal scales as demonstrated by Eke et al. (2006) for fluctuating cerebral blood volume in humans and Herman et al. (2011) for resting-state fMRI BOLD signals in rat. As shown in the frequency domain by spectral analysis, in both species, scale-free structuring of the signal was present across a range of frequencies well below the Nyquist frequency (half of the sampling frequency). It was characterized by a systematically and self-similarly increasing power toward lower frequencies that could be modeled by Eq. 25 yielding a spectral index of β > 0, which is an indication of serial correlation between the temporal events (long-term memory). Above this range, the fluctuations were found random with β ≈ 0 meaning that subsequent temporal events were not correlated. The separation of these ranges therefore is crucial because failing to do so would cause a bias in the estimate of β.

For fractal time series analysis a proper scaling range should be selected where fluctuations are scale-invariant. Optimization of the sampling process, as well as the regression analysis on log-log representations of measures vs. scales yielding the scaling exponent is essential (Eke et al., 2002). In case of time domain methods such as DFA, DMA, and AFA as well introduced by Gao et al. (2011), optimizing the goodness-of-fit of the regression analysis is an example. Detailed recommendations as to how to deal with this problem can be found elsewhere (Peng et al., 1994; Cannon et al., 1997; Eke et al., 2002; Gao et al., 2006). When a signal’s spectrum contains other than monofractal components, it may prove difficult to select a monofractal scaling range even by isolating local scaling ranges and fitting local slopes for the spectral index. This procedure should be carefully carried out given that local ranges may end up containing inadequately few spectral estimates for a reliable fitting of the trendline. When the aim is to assess the topology of the measure, this criterion can be relaxed (Herman et al., 2011).

Faghfouri and Kinsner (2005) reported that improper selection of scaling range has a detrimental effect on the results of WTMM. Different scales correspond to different window sizes in MF-DFA and MF-DMA method, and discarding the smallest and largest window sizes was even suggested by Peng et al. (1994) for the original DFA. Cannon et al. (1997) and Gao et al. (2006) suggested an optimization for the appropriate range of analyzing window sizes (i.e., scales). While this can be regarded as best practice in carrying out MF-DFA, some degree of bias is still introduced to the results arising mainly from the smallest window sizes (Bryce and Sprague, 2012).

#### Dualism in multifractal formalism

Amongst the indirect, moment-based methods, WTMM uses a different approach to obtain the singularity spectrum than MF-DFA and MF-DMA. Convergence of this dualism is very unlikely, as the relationship of exponents in MF-DFA to the multifractal formalism is reported to be valid only in special cases (Yu and Qi, 2011). The seminal paper of MF-DFA Kantelhardt et al. (2002) established a relationship between the generalized Hurst exponent and multiscaling exponent. The validity of this equation was reported to be valid only if *H* = 1 (Yu and Qi, 2011), and thus another derivation for τ(*q*) was proposed. In addition, singularity spectra reported with MF-DFA – as it follows from the Legendre transform of τ(*q*) (Eq. 9) – always reaches their maxima at 1, while this does not hold for wavelet methods. In our opinion, revision of results obtained with MF-DFA may be necessary along with consolidating the multifractal formalism published in the field, using the original papers as a starting point of reinvestigation (Frisch and Parisi, 1985; Mandelbrot, 1986; Barabási and Vicsek, 1991; Muzy et al., 1993; Arneodo et al., 1998).

## Demonstration

Scrutinizing relevant data in selected previous works recognized as having proven or potential impact on the development of the field will likely demonstrate some typical pitfalls.

### Significance of system noise in the interpretation of fMRI BOLD fluctuations

Zarahn et al. (1997) demonstrated early in a careful analysis on spatially unsmoothed empirical human fMRI BOLD data (collected under null-hypothesis conditions) that the examined datasets showed a disproportionate power at lower frequencies resembling of 1/*f* type noise. In spite of the very detailed analysis, these authors treated the 1/*f* character as a semi-quantitative feature of fMRI noise and accepted its validity over a decaying exponential model as the form of the frequency domain description of the observed intrinsic serial, or autocorrelation. The spectral index, β, however was not reported but can be reconstructed from the power slope by converting the semilog plot of power vs. frequency in their Figure 3D panel to a log-log plot compatible to |*A*(*f* ) |^2^∝1/*f* ^β^ model. A β value of ∼3.3 is yielded, which is exceedingly higher than the values of 0.6 < β < 1.2 reported recently for an extensive 3T dataset by He (2011). This precludes the possibility that the collected resting-state 1.5T BOLD dataset would have been of physiological origin. Our recently reported results for the rat brain with −0.5 < β < 1.5 reconfirms this assertion (Herman et al., 2011). In fact, Zarahn et al. (1997) wished to determine if the 1/*f* component of the noise observed in human subjects was necessarily due to physiological cause, but had to reject this hypothesis because they found no evidencing data to support this hypothesis. Zarahn et al. (1997) felt the AC structure (in the time domain, which is equivalent to the inverse power law relationship in the frequency domain) may not be the same for datasets acquired in different magnets, not to mention the impact of using various fMRI scanning schemes (Zarahn et al., 1997). Accordingly, and in light of our rat data for magnets 4, 9.4, and 11.7T, a less than optimal field strength could have led to a signal definition inadequate to capture the 1/*f*^β^ type structuring of the BOLD signal of biological origin that must have been embedded in the human datasets Zarahn et al. (1997) but got overridden by system noise. Most recently, Herman et al. (2011) and He (2011) referred to the early study of Zarahn et al. (1997) as one demonstrating the impact of system noise on fMRI data, while Fox et al. (2007) and Fox and Raichle (2007) as the first demonstration of 1/*f* type BOLD noise with the implication that the 1/*f* pattern implied fluctuations of biological origin.

### Significance of the general 1/f^β^ vs. the strict 1/f model in the interpretation of fMRI BOLD noise data

Fox et al. (2007) reported on the impact of intrinsic BOLD fluctuations within cortical systems on inter-trial variability in human behavior (response time). In conjecture of the notion that the variability of human behavior often displays a specific 1/*f* frequency distribution with greater power at lower frequencies, they remark “This observation is interesting given that spontaneous BOLD fluctuations also show 1/*f* power spectrum (Figure S4). While the 1/*f* nature of BOLD fluctuations has been noted previously (Zarahn et al., 1997), we show that the slope is significantly between −0.5 and −1.5 (i.e., 1/*f* ) and that this is significantly different from the frequency distribution of BOLD fluctuations observed in a water phantom,” and in their Figure S4 conclude that “the slope of the best fit regression line (red) is −0.74, close to the −1 slope characteristic of 1/f signals.” This interpretation of the findings implies that the spontaneous BOLD fluctuations can be adequately described by the “strict” 1/*f* model, where the spectral index, β, in 1/*f* ^β^ – known as the “general” inverse power law model – is treated as a constant of 1, not a variable carrying information on the underlying physiology. Incidentally, studies of Gilden and coworkers (using a non-fMRI approach) have indeed demonstrated (Gilden et al., 1995; Gilden, 2001; Gilden and Hancock, 2007) that response time exhibits variations that could not be modeled by a strict 1/*f* spectrum but by one incorporating a varying scaling exponent (Gilden, 2009).

Scrutinizing the data of Figure S4 can offer an alternative interpretation as follows. In terms of the hardware, the use of 3T magnet must have ensured adequate signal definition for the study. In their Figure S4, spectral slopes were reported in a lumped manner, in that power at each and every frequency were averaged for the 17 human subjects first (thus creating frequency groups), and then mean slopes along with their statistical variation were plotted for the frequency groups. The mean slope of −0.74 (of thee lumped spectrum) was obtained by regression analysis. This treatment of the data implies that the |*A*(*f* )|^2^ ∝ 1/*f* ^β^ model (Mandelbrot and Ness, 1968; Eke et al., 2000, 2002) was *a priori* rejected otherwise the slope should have been determined for each and every subject in the group across the range of observed frequencies and their associated power estimates (of the true spectrum) first, followed by the statistical analysis for the mean and variance within the group of 17 subjects, for the following reasons. The spectral index is found by fitting a linear model of |*A*(*f* )|^2^ ∝ 1/*f* ^β^ across spectral estimates for a range of frequencies. In our opinion when it comes to provide the mean spectral index, it is indeed reasonable (Gilden and Hancock, 2007; Gilden, 2009) to come up with statistics on the fractal estimates for a group of time series data first by obtaining the estimates, proper. Averaging spectral estimates at any particular frequencies and assembling an average spectrum from them tend to abolish the fractal correlation structure for any particular time series and develop one for which the underlying time series is indeed missing. Because the transformation between the two treatments is not linear, the true mean slope of the scale-free analysis cannot be readily reconstructed from the reported slope of the means. Nevertheless, if we regard its value as an approximation and convert it to β, which being less than 1 warrants the use of *H*′ = (β_fGn_ + 1)/2, one would yield a value of β = 0.77 and *H*′ = 0.87, respectively.

A recent review by Fox and Raichle (2007) offers an impressive overview and insight of how to delineate cooperative areas (or systems) in the brain based on functional connectivity that emerges from spatial cross-correlation maps of regional fluctuating BOLD signals in the resting brain (Biswal et al., 1995). These authors place the spontaneous activity of the brain as captured in BOLD fluctuations in spatio-temporal domains of fMRI data in the focus of the review emphasizing that itis a fingerprint of a newly recognized mode of functional operation of the brain referred to as default or intrinsic mode (Fox and Raichle, 2007). They argue that the ongoing investigation of this novel aspect of the mode of brain’s operation using fractal analysis of resting-state fMRI BOLD may lead to a deeper and better understanding of the way the brain – on the expense of very high baseline energy production and consumption by glucose and oxidative metabolism – maintains a mode capable of selecting and mobilizing these systems in order to respond to a task adequately (Hyder et al., 2006). One has to add that the default or intrinsic mode of operation has been demonstrated and investigated in overwhelming proportions by connectivity analyses based on cross-correlating BOLD voxel-wise signals as opposed on AC of single voxel-wise BOLD time series.

Fox and Raichle (2007) emphasize “spontaneous BOLD follows a *1/f distribution*, meaning that there is an increasing power in the low frequencies.” In their furthering on the nature of this 1/*f* type distribution they refer to the studies of Zarahn et al. (1997) and Fox et al. (2007) in the context it was described above (Fox et al., 2007) reaching the same conclusion, in that the characteristic model of human spontaneous BOLD is the 1/*f* (meaning the “strict”) model. We would like to suggest that the notion of *1/f distribution* having a regression slope of close to −1 on the log-log PSD plot is somewhat misleading.

In an attempt to consolidate this issue, we suggest that the data be fitted to a model in the form of 1/*f*^β^, where β is a variable (Eke et al., 2000, 2002) responding to states of physiology (Thurner et al., 2003; He, 2011) of characteristic topology (Thurner et al., 2003; Herman et al., 2011) in the brain, not a constant of 1. A potential advantage of this model is that by regarding β as a scaling exponent the distribution can then be described to be scale-free (or fractal).

### Significance of the 1/f^β^model and the dichotomous fGn/fBm analytical strategy in analyzing scaling laws and persistence in human brain activity

As seen above, from the modeling point of view the issue of a reliable description of the autoregressive signal structuring of spontaneous BOLD, is fundamental and critical in resting-state. If it is done properly, it can lend a solid basis for assessing changes in the scaling properties in response to changing activity of the brain. The study of Thurner et al. (2003) was probably the first to demonstrate that spontaneous BOLD in the brain was scale-free and that the scaling exponent of inactive and active voxels during sensory stimulation differed. At the time of the publication of their study, the monofractal analytical strategy of Eke et al. (2000, 2002) based on the dichotomous fGn/fBm model of Mandelbrot and Ness (1968) did not yet reached the fMRI BOLD community, hence Thurner et al. (2003) did not rely on it, either. In this section we will demonstrate the implications of this circumstance in terms of the validity and conclusions of their study. We will do it in a detailed, didactical manner so that our reader should gain a hands-on experience with the perplexed nature of the issue.

Subtracting the mean from the raw fMRI signal precedes the analysis proper, ${Ī}^{\vec{x}}$(*t*), yielding $I^{\vec{x}}$(*t*) in Eq. 39,

(39) $$I^{\vec{x}}\left(t\right)={Ī}^{\vec{x}}\left(t\right)-{\left\langle {Ī}^{\vec{x}}\left(t\right)\right\rangle }_{t},$$

which step is compatible with (D)FA (Eke et al., 2000).

Subsequently, in Eq. 40, the temporal correlation function, $C^{\vec{x}}\left(\tau \right)$, is calculated

(40) $$C^{\vec{x}}\left(\tau \right)=\left\langle I^{\vec{x}}\left(t\right)I^{\vec{x}}\left(t+\tau \right)\right\rangle =\frac{1}{N-\tau }\overset{N-\tau }{\underset{t=1}{\sum }}I^{\vec{x}}\left(t\right)I^{\vec{x}}\left(t+\tau \right).$$

In fact in this step of the analysis the covariance was calculated given that a division by variance was missing. Hence, it is slightly misleading to regard Eq. 40 as the temporal (or auto) correlation (see Eke et al., 2000, Eq. 2). Only, if assumed that the signal is fGn, whose variance is known to be constant over time, the covariance function can be taken as equivalent to the AC function. Because the authors have not tested and proven the signal’s class was indeed fGn (Eke et al., 2000), there is no basis for the validity of this assertion.

In Eq. 41, the signal is summed yielding $X_{n}^{\vec{x}}\left(\tau \right),$ in order to eliminate problems in calculating the AC function due to noise, non-stationarity trends, etc.

(41) $${X_{n}}^{\vec{x}}\left(\tau \right)=\overset{n}{\underset{t=1}{\sum }}I^{\vec{x}}\left(t\right)$$

This form of the signal is further referred to as “voxel-profile.”

Note, that the signal remains in this *summed* form for the rest of the analysis (i.e., analyzed as fBm). As a consequence, spectral analysis later in the study was applied to a summed – hence *processed* – signal and the results were thus reported for this and not the raw fMRI signal, which circumstance prevented reaching a clear conclusion.

Furthermore, the authors indicated that the temporal correlation function would characterize persistence. It seems the two terms (correlation vs. persistence) are used as synonyms of one another whereas they are not interchangeable terms: persistence is a property of fBm, while correlation is that of an fGn signal (Eke et al., 2000). Please note, as the raw signal has been summed, the covariance here characterized persistence that was not present in the raw fMRI signal.

In the next step (Eq. 42), the AC function is approximated by a power law function with γ as its exponent

(42) $$C^{\vec{x}}\left(\tau \right)\sim \tau^{-\gamma },\cdots 0<\gamma <1.$$

Based on the equation of the AC function using the Hurst exponent, *H*, γ must be proportional to 2*H* (Eke et al., 2000, Eq. 15).

Subsequently, as a part of a FA of the authors (cited in their Reference 19 as unpublished results of their own), the statistics ($F^{\vec{x}}\left(\tau \right),$ standard deviation) was calculated for the AC function in Eq. 43

(43) $$F^{\vec{x}}\left(\tau \right)={\left\langle {\left(X_{n+\tau }^{\vec{x}}-X_{n}^{\vec{x}}\right)}^{2}\right\rangle }_{n}^{1/2}.$$

In the left side of Eq. 44, a general power law was applied to the fluctuation from Eq. 43 as $F^{\vec{x}}\left(\tau \right)\sim \tau^{\alpha }$

(44) $$F^{\vec{x}}\left(\tau \right)\sim \tau^{\alpha },\;\alpha =1-\gamma /2.$$

(Note, as the fluctuations have not been detrended, this method is not the DFA of Peng et al., 1994 but strongly related to it).

Consider the scaling exponent, α, on the left side of Eq. 44. According to Peng et al. (1994) and Eke et al. (2002) α = *H* only if the raw signa l,$I^{\vec{x}}\left(t\right),$ is an fGn. However, because at this point the summed raw signal, $X_{n}^{\vec{x}}\left(\tau \right),$ is the object of the analysis, α and *H* should relate to each other as α = *H* + 1. Given that the signal was summed in Eq. 41 leading up to Eq. 43, and the values for “outside the brain” were reported as α ≈ 0.5, and for “inside the brain” as 0.5 < α < 1, α must have been improperly calculated because α cannot possibly yield a value of 0.5 for a summed signal given that *H* scales between 0 and 1 and for an fBm series α = *H* + 1 holds. The reported value of 0.5 < α < 1 can be regarded correct only for $I^{\vec{x}}\left(t\right),$ the raw fMRI signal, which therefore had to be an fGn process. On the other hand, the reported values 2 < β < 3 are correct for the $X_{n}^{\vec{x}}$ signal, only (for reasons given later). Hence the reported α and β values lacking an indication of their respective signal class ended up being ambiguous.

Next, consider the right side of Eq. 44, which expresses α by using γ introduced earlier. We just pointed out that the raw fMRI signal must have been an fGn with α ≡ *H*. Consequently, α can be substituted for *H* in Eq. 44 as *H* = 1 − γ/2 and γ expressed as

(45) γ=2-2H.

The authors referring to power law decays in the correlations relate the spectral index, β, to γ as

(46) β=3-γ,

and further to α as

β=2α+1.

Note, that these relations between β, γ, and α in principle do depend on signal class that was not reported.

Now, let us substitute γ as expressed in Eq. 45 into Eq. 46

β=3-2+2H=1+2H,

then express *H*

(47) $$H=\frac{\beta -1}{2}.$$

As shown by Eke et al. (2000), Figure 2; in Eke et al. (2002), Table 1, based on the dichotomous fGn/fBm model, Eq. 47 would have equivocally identified the case of an fBm signal. As pointed out earlier, the raw fMRI signal was summed before the actual fractal analysis. Consequently, the relationship β = 3 – γ ends up holding only if the raw fMRI signal was an fGn process. This is therefore the second piece of evidence suggesting that the class of the raw fMRI signal must have been fGn. Nevertheless, the relationship β = 2α + 1 could not hold concomitantly for reasons that follow. In an earlier paper of the group (Thurner et al., 2003), the authors stated “The relationship is ambiguous, however, since some authors use the formula α = 2*H* + 1 for all values of α, while others use α = 2*H*−1 for α < 1 to restrict *H* to range (0,1). In this paper, we avoid this confusion by considering α directly instead of *H*.” The fGn/fBm model (Eke et al., 2002) helps resolving this issue as neither of these relationships between α and *H* holds because if α is calculated with the signal class recognized and determined, the relationship between α and *H* is equivocally α = *H*_fGn_ and α = *H*_fBm_ + 1. Based on the fGn/fBm model, the relationship between β and α given in Eq. 46 as β = 2α + 1 needs to be revised, too, to is correct form of β = 2α − 1 (See Table 1 in Eke et al., 2002).

Thurner et al. (2003) concluded: “Outside the brain and in non-active brain regions voxel-profile activity is well described by classical Brownian motion (random walk model, α ∼ 0.5 and β ∼ 2).” Recall, the “voxel-profile” is not the raw fMRI signal (intensity signal, $I^{\vec{x}}\left(t\right),$ most probably an fGn), but its summed form, $X_{n}^{\vec{x}}\left(\tau \right),$ an fBm.

Our conclusion on the above analysis by Thurner et al. (2003) is as follows: (i) α was improperly calculated by the authors’ FA method because α ∼ 0.5 cannot possibly be valid for an fBm signal given that α_fBm_ > 1 (Peng et al., 1994), (ii) β ∼ 2 is only formally valid given that it was calculated based on Eq. 46 from an improperly calculated α and by using an arbitrary relationship between α and β. The subsequent and opposite effects of these rendered the value of β to β ∼ 2.

When the results of Thurner et al. (2003) are interpreted according to the analytical strategy of Eke et al. (2000) based on the dichotomous fGn/fBm model of Mandelbrot and Ness (1968), the reported values of Thurner et al. can be converted for their fMRI “voxel-profile” data $X_{n}^{\vec{x}}$ to α_fBm_ ∼ 1.5, β_fBm_ ∼ 2, *H*_fBm_ ∼ 0.5 or for the raw fMRI intensity signal $I^{\vec{x}}\left(t\right)$ to α_fGn_ ∼ 0.5, β_fGn_ ∼ 0, *H*_fGn_ ∼ 0.5. This interpretation of the data reported for humans by Thurner et al. (2003) is fully compatible with the current findings by He (2011) on the human and by Herman et al. (2009, 2011) on the rat brain.

### Multifractal analyses on rat fMRI BOLD data

Exemplary analysis on empirical BOLD data is presented on the 11.7T coronal scan shown in Figure 10 to demonstrate the inner workings of these methods when applied to empirical data, and point to potential artifacts, too (See Figure 12). For monofractal analysis, we recommend using monofractal SSC for it gives unbiased estimates across the full range of the fGn/fBm dichotomy. For this reason, the topology is well defined and not as noisy as on the PSD maps. MF-DFA, due to its inferior performance in the strongly correlated fGn range (See Figure 6 at *q* = 2), failed with this particular BOLD dataset. Also note, that the histograms obtained for the same datasets evaluated by these different methods do differ indicating that method’s performance were different. Proper interpretation of the data therefore assumes an in-depth understanding of the implication of method’s performance on the analysis. *P*_c_ and most certainly *W* seems a promising parameter to map from the BOLD temporal datasets. Their proper statistical analyses along with those of singularity spectra for different anatomical locations in the brain should be a direction of future research.

## Physiological Correlates of Fractal Measures of fMRI BOLD Time Series

Eke and colleagues suggested and demonstrated that β should be regarded as a variable responding to physiology (Eke et al., 1997, 2000, 2002, 2006; Eke and Herman, 1999; Herman and Eke, 2006; Herman et al., 2009, 2011).

Soon, Bullmore et al. (2001) suggested treating 1/*f* type fMRI BOLD time series as realizations of fBm processes for the purpose of facilitating their statistical analysis using pre-whitening strategies. For this reason, signal classification did not emerge as an issue to address. Then Thurner et al. (2003) demonstrated that human resting-state fMRI BOLD is not only a scale-free signal, but do respond to stimulation of the brain. Their analysis yielded this conclusion in a somewhat arbitrary manner in that the importance of the fGn/fBm dichotomy was not recognized at the time that led to flaws in the calculation of the scaling exponent as demonstrated above. Hu et al. (2008) and Lee et al. (2008) also reported that *H* obtained by DFA can discriminate activation from noise in fMRI BOLD signal.

In later studies dealing with the complexity of resting-state and task-related fluctuations of fMRI BOLD, the issue of signal class has gradually shifted into the focus (Maxim et al., 2005; Wink et al., 2008; Bullmore et al., 2009; He, 2011; Ciuciu et al., 2012).

Recently Herman et al. (2011) found in the rat brain using PSD that a significant population of fMRI BOLD signal fell into the non-stationary range of β. The inference of this finding is the potential interference of non-stationary signals with resting-state connectivity studies using spatio-temporal volumes of fMRI BOLD. It is even more so, if SSC is used for signal classification (Figure 11) and analysis (Figure 12) shifting the population histogram of *H*′ to the right.

The β value converted from the reported human spectral slopes by Fox et al. (2007) (see above) fits very well within the range of human data reported most recently by He (2011) for the same instrument (3T Siemens Allegra MR scanner). He (2011) adopting the dichotomous monofractal analytical strategy of Eke et al. (2002) demonstrated that β of spontaneous BOLD obtained for multiple regions of the human brain correlates with brain glucose metabolism, a fundamental functional parameter offering grounds for the assertion that that β itself is a functional parameter. Herman et al. (2011) using the same analytical strategy (Eke et al., 2000, 2002) on resting-state rat BOLD datasets showed that β maps capture a gray vs. white matter topology speaking for the correlation of β and functional activity of the brain regions being higher in the gray than in the white matter.

With near infrared spectroscopy, – recommended by Fox and Raichle (2007) as a cost-effective, mobile measurement alternative of fMRI to capture resting-state hemodynamic fluctuations in the brain – a 1/*f* ^β^ temporal distribution of cerebral blood volume (one of the determinant of BOLD) was found in humans, with an age and gender dependence on β (Eke et al., 2006). Furthermore, β determined from heart rate variability time series was found to differ between healthy and unhealthy individuals (Makikallio et al., 2001).

The above physiological correlates seem to have opened a new perspective in basic and clinical neurosciences (Hausdorff et al., 1997) by recognizing β as an experimental variable and applying adequate tools for its reliable assessment (Pilgram and Kaplan, 1998; Eke et al., 2000, 2002; Bullmore et al., 2009; He, 2011) with multifractal analyses as a dynamically expanding perspective (Ciuciu et al., 2012; Ihlen, 2012), too.

We propose that the inter-regional spatial cross-correlation (connectivity) as a means of revealing *spatial organization* in the brain be supplemented by a temporal AC analysis of extended BOLD signal time series by mapping β as an index of *temporal organization* of the brain’s spontaneous activity.

## Conflict of Interest Statement

The authors declare that the research was conducted in the absence of any commercial or financial relationships that could be construed as a potential conflict of interest.
